# Despite structural identity, *ace-1* heterogenous duplication resistance alleles are quite diverse in *Anopheles* mosquitoes

**DOI:** 10.1038/s41437-024-00670-9

**Published:** 2024-01-27

**Authors:** Jean-Loup Claret, Marion Di-Liegro, Alice Namias, Benoit Assogba, Patrick Makoundou, Alphonsine Koffi, Cédric Pennetier, Mylène Weill, Pascal Milesi, Pierrick Labbé

**Affiliations:** 1 ISEM, Université de Montpellier, CNRS, IRD, Montpellier, France; 2 Medical Research Council, Unit The Gambia at London School of Hygiene and Tropical Medicine, London, UK; 3 National Institute of Public Health/Pierre Richet Institute, Bouake, Côte d’Ivoire; 4 Institute of Research for Development (IRD), Marseille, France; 5 Plant Ecology and Evolution, Department of Ecology and Genetics, Uppsala University, Norbyvägen 18D, 75236 Uppsala, Sweden; 6 Science for Life Laboratory (SciLifeLab), Uppsala University, 75237 Uppsala, Sweden; 7 Institut Universitaire de France (IUF), Paris, France

**Keywords:** Evolutionary genetics, Genome evolution

## Abstract

*Anopheles gambiae s.l*. has been the target of intense insecticide treatment since the mid-20th century to try and control malaria. A substitution in the *ace-1* locus has been rapidly selected for, allowing resistance to organophosphate and carbamate insecticides. Since then, two types of duplication of the *ace-1* locus have been found in *An. gambiae s.l*. populations: homogeneous duplications that are composed of several resistance copies, or heterogeneous duplications that contain both resistance and susceptible copies. The substitution induces a trade-off between resistance in the presence of insecticides and disadvantages in their absence: the heterogeneous duplications allow the fixation of the intermediate heterozygote phenotype. So far, a single heterogeneous duplication has been described in *An. gambiae s.l*. populations (in contrast with the multiple duplicated alleles found in *Culex pipiens* mosquitoes). We used a new approach, combining long and short-read sequencing with Sanger sequencing to precisely identify and describe at least nine different heterogeneous duplications, in two populations of *An. gambiae s.l*. We show that these alleles share the same structure as the previously identified heterogeneous and homogeneous duplications, namely 203-kb tandem amplifications with conserved breakpoints. Our study sheds new light on the origin and maintenance of these alleles in *An. gambiae s.l*. populations, and their role in mosquito adaptation.

## Introduction

Human activities have huge impacts on the environment, these diverse anthropogenic modifications can lead to spectacular adaptations in species subjected to them (see for example Otto 2018 or Hendry et al. 2017). Among these, resistance to biocides (e.g. antibiotic resistance in bacteria or resistance to pesticides in crop pests and disease vectors) are probably the most studied and the best understood, because of their crucial impacts on economy and public health. From an evolutionary biology point of view, these are also major models to decipher the genetics of the adaptation (e.g. polygenic *vs* mono- or oligo-genic) or to understand how evolutionary processes shape these dynamics (e.g. spatial variation in selective pressure intensity, fluctuating selection overtime; Guillemaud et al. 1998; David et al. 2010; Milesi et al. 2016). Notably, studying resistance revealed the complexity and the diversity of the genomic structural rearrangements underlying adaptations well beyond the role of single nucleotide polymorphisms (SNPs). For example, there are many studies that link gene duplications to cases of xenobiotic resistance (Devonshire and Sawicki 1979; Leister 2004; Labbé et al. 2007; Kwon et al. 2010; Patterson et al. 2018). In the present study, we focused on one of these well-known models, the case of insecticide resistance in the malaria-vector mosquito *Anopheles gambiae s.l*., and on the genomic nature and diversity of resistance alleles at the *ace-1* locus.

*Anopheles gambiae s.l*. has been the target of intense insecticide treatment since the mid-20^th^ century, particularly on the African continent, to control malaria. While pyrethroids (PYR) are the most used insecticides, organophosphates (OP) and carbamates (CX) have also been utilised since the 1950’s. They target acetylcholinesterase (AChE), an enzyme that regulates the activity of the synaptic neurotransmitter acetylcholine (Weill et al. 2003). A unique substitution in the AChE-encoding gene *ace-1*, resulting in a glycine to serine substitution at the 280^th^ codon of the protein (G280S). The substitution enables resistance to OP/CX by hindering their binding to AChE (R allele, Weill et al. 2004), often referred to as the G119S mutation according to its position in the homologous gene of *Torpedo californica*, where AChE structure was first elucidated (Schumacher et al. 1986). We adopt this nomenclature here. This mutation has been independently selected for in multiple mosquito species (Weill et al. 2003; Huchard et al. 2006). However, the enzymatic activity of the protein encoded by the R allele is 60% lower than its wild-type susceptible counterpart (S allele; Bourguet et al. 1997; Alout et al. 2008; Labbé et al. 2014). As a result, the R alleles are selected against in the absence of OP/CX insecticides. So far in *An. gambiae s.l*., G119S is the only SNP mutation that has been found responsible for OP/CX insecticide resistance, introgressed from *An. coluzzii* to *An. gambiae s.s*. (Weill et al. 2003; Djogbénou et al. 2008; Assogba et al. 2016; Grau-Bové et al. 2021; a couple of other mutations have however been found in other mosquito species, Alout et al. 2007a, 2007b).

**Fig. 1:**
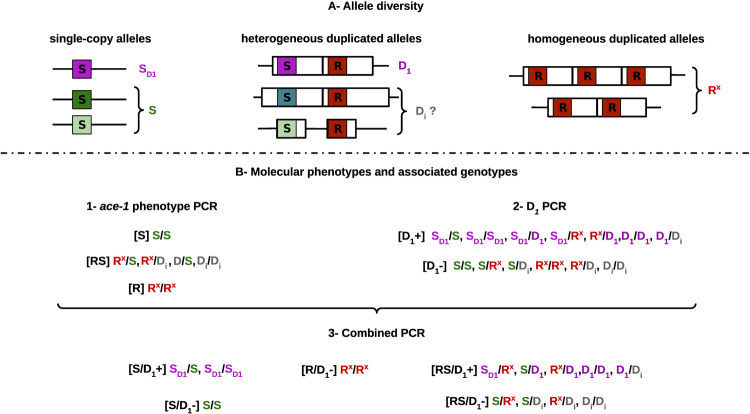
Diversity of *ace-1* alleles, and molecular phenotyping. (Modified from Assogba et al. 2018). **A** The various alleles found at the *ace-1* locus: different single-copy S alleles^a^ on the left (green) and homogeneous duplicated alleles R^x^ on the right (here with 2 or 3 R copies, R^3^ and R^2^ resp., in red). The central part illustrates the known D_1_ heterogenous allele, with its D(S) copy (in pink) and its D(R) copy (in red), as well other heterogeneous D_i_ alleles (with different architectures depending on the size of the amplified region). NB: the single-copy S_D1_ allele has the same sequence as the D(S) copy of *ace-1* (hence the same colour). **B** The two PCR used to identify the genotypes of triple peaks samples. The combined information of the « *ace-1* phenotype » PCR (1) and the «D_1_ PCR» (2) allow the partial discrimination of 5 phenotypes with 13 possible genotypes (3). ^a^multi-copy S alleles or S^x^ could also exist, but they would not be distinguished here from single-copy alleles.

Other types of mutations, i.e. structural variants (SVs), have also been selected in response to the use of these insecticides. Two types of duplication of the *ace-1* locus have been found in *An. gambiae s.l*. (Fig. 1A): i) homogeneous duplications, i.e. composed of several R copies (R^x^ alleles; Assogba et al. 2016; Grau-Bové et al. 2021), or ii) heterogeneous duplications, containing both R and S copies (D alleles; Assogba et al. 2015, 2016; Grau-Bové et al. 2021). There is a trade-off between resistance in the presence of insecticides and disadvantage in their absence (or “selective cost”, but see Lenormand et al. 2018): R^x^ alleles confer higher resistance levels and are favoured in highly-treated areas, but are associated with stronger disadvantages in absence of insecticides. The heterogeneous duplications (D alleles) enable the fixation of the heterozygous phenotype, i.e. intermediate levels of both resistance and selective disadvantage (Labbé et al. 2014; Assogba et al. 2015; Milesi et al. 2017). While R^x^ and S remain respectively the fittest alleles in highly-treated and non-treated areas, D alleles are the fittest in populations exposed to intermediate selective pressures, in those areas exposed to reduced concentrations of treatments per se, or to temporal or geographical variations in treatment intensity (Labbé et al. 2014; Milesi et al. 2017).

Diversity in duplicated alleles (and more generally in copy-number variations or CNV) can result from two types of variation: i) variation in the DNA sequence of the amplicons, in particular the *ace-1* haplotypes, and/or ii) copy-number variations (for example R^x^ alleles carry different copy-numbers of the same haplotype). In the present study, we focussed on haplotype variations: we will refer to alleles carrying different *ace-1* haplotypes as “sequence-alleles”, and those differing in number of copies as “copy-number-alleles”. In *An. gambiae s.l*., a single R sequence-allele, but with multiple copy-number-alleles or R^x^ alleles, was found in several African countries (Assogba et al. 2016). Similarly, only one D sequence-allele, named Ag-D_1_ (thereafter D_1_ for simplicity), has been formally described, using direct sequencing of cloned fragments of the *ace-1* gene (real haplotypes, Djogbénou et al. 2008). D_1_ carries two *ace-1* copies, one R copy (the haplotype is identical to that found in R^x^ alleles) and one S copy, in 203-kb tandem amplicons (Assogba et al. 2016). D_1_ is found all over West Africa (Assogba et al. 2018), which is in sharp contrast with *Cx. pipiens*, where several different D sequence-alleles are often found segregating in the same population (Milesi et al. 2018). Grau-Bové et al. (2021) recently analysed a large dataset of Illumina paired-end genomes of *An. gambiae s.l*. from all over Africa (The *Anopheles gambiae* 1000 Genomes Consortium (2021): Ag1000G phase 3). Based on the variations in depth of coverage of the alternative bases at position 119 (i.e. of R or S sequences), they suggested that several copy-number-alleles, differing in their numbers of R and S copies, might actually be segregating in Africa, while the nature of their data (i.e. short-read sequencing) was unreliable to assess whether multiple D sequence-alleles were segregating in the African populations.

Evidence for the existence of diversity in D alleles could help us understand the origin of these mutations, and more generally how SVs are selected for short-term adaptation. So, we adopted a comprehensive approach to assess the *ace-1* haplotype diversity and the number of R and S copies in heterogeneous duplications and used information from various sources to understand the origin of this diversity. As previous studies have shown that the frequency of D alleles in *An. gambiae s.l*. was particularly high in Ivory Coast (Assogba et al. 2018), and as Grau-Bové et al. (2021) suggested that several D copy-number-alleles could segregate there, we analysed the structures and diversity of the heterogeneous duplications present in two natural populations of Ivory Coast, Yamoussoukro and Yopougon. By screening samples from Assogba et al. (2018), we found that the presence of the D_1_ allele alone could not explain the observed frequencies of D alleles. By cloning and sequencing a large part of the *ace-1* locus for several individuals, we obtained the various haplotypes of each of their D(R) and D(S) copies. We also tested a more recently developed, and logistically easier approach, based on long-read sequences of PCR products (Namias et al. 2023). We revealed that at least nine different *ace-1* D alleles segregate in these two populations. Using whole genome sequencing, we showed that at least five of these alleles share the exact same structure, two 203-kb tandem amplicons, with the exact same breakpoints. Finally, we discuss what these findings suggest in terms of duplication origin, but also the role of SVs in the adaptation process.

## Material and methods

### Sampling and identification

We focused on two localities from Ivory Coast where the presence of the D_1_ heterogeneous duplication has already been documented (Assogba et al. 2018; Grau-Bové et al. 2021), and where resistance has been monitored since 2012. We first used DNA previously extracted from adult mosquito samples, collected in 2012, 2015, 2016, and preserved at −80 °C in the lab (Assogba et al. 2018). To assess the frequencies at the time of the study we collected new fourth instar (L_4_) larvae samples in the same sites in 2019. They were identified as *An. coluzzii* through a multiple PCR protocol: the first PCR was able to discriminate *An. arabiensis* from *An. gambiae s.s*. and *An. coluzzii* (Supporting information Table 1, “Species”; Scott et al. 1993), and a second one distinguishing *An. gambiae s.s*. from *An. coluzzii* (Supp. Info. Table 1, “Form”; Favia et al. 1997).

### DNA extraction and PCR conditions

We extracted DNA from individual L_4_ following a protocol modified from Collins et al. (1987). Briefly, each larva was ground in 200 µL CTAB buffer (100 mM Tris HCL, pH8.0, 10 mM EDTA,1.4 M NaCl, 2% CTAB), then incubated for 15 min, at 60 °C. 200 µL of chloroform with 4% of isoamyl alcohol were added and the solution was centrifuged for 10 min at 8000 rotations/min. The supernatant was transferred to a new tube with 200 µL of isopropanol to precipitate DNA at room temperature. DNA was washed with 400 µL of 70% ethanol after 10 min of centrifugation (10,000 rotations/min), dried, and then rehydrated in 50 µL H_2_O.

The PCR tests described below were performed using the Promega PCR kit (Madison, Winsconsin, USA) with *ca*. 50 ng of genomic DNA into 40 μL of PCR-mix and using the following: 94 °C for 30 s, annealing temperature for 30 s, and 72 °C for 1 to 2 min for a total of 30 cycles (primers and annealing temperatures are listed in Supp. Info. Table 1).

### Heterogeneous duplication detection and frequency estimation

#### *ace-1* phenotyping

We performed the “*ace-1* phenotype” PCR-RFLP test described in Djogbénou et al. (2008): it amplifies an 817 bp sequence of the *ace-1* locus encompassing the resistance-diagnostic G119S mutation. This mutation generates an *Alu I* restriction site and enables the distinction between three phenotypes: resistant homozygous [RR], susceptible homozygous [SS], and heterozygous [RS] (Fig. 1B-1). However, it does not enable the differentiation between standard heterozygous individuals for single-copy alleles (RS) and individuals carrying a heterogeneous duplicated allele (D), as D alleles associate both susceptible D(S) and resistant D(R) haplotypes of the *ace-1* locus (Assogba et al. 2015). Alleles with multiple identical copies (e.g. R^x^) also cannot be distinguished from alleles carrying the same haplotype but as single-copy.

#### Estimation of D allele frequencies

D allele frequencies can nevertheless be inferred from the phenotypic frequencies (Table 1), as their presence in a population causes an excess of heterozygous genotypes for the R and S alleles relative to what is expected under Hardy-Weinberg equilibrium (Lenormand et al. 1998). We took advantage of this observation and used the same approach as in Assogba et al. (2018) to compute the D allele frequencies, independently for each year and location, implementing the maximum likelihood approach developed by Lenormand et al. (1998). We calculated the log-likelihood, *L*, of observing all the data as follow:

$$L={\sum }_{i}{n}_{{ijt}}\mathrm{ln}({f}_{{ijt}})$$

with *n*_*ijt*_ and *f*_*ijt*_, the observed number and the predicted frequency of individuals with phenotype *i* in population *j* at time *t*, respectively. *L* was maximised independently for each sample (i.e. population *j* at time *t*) using a simulated annealing algorithm (Labbé et al. 2009; Lenormand et al. 1998). The support limits (SL, equivalent to 95% confidence intervals) were defined as the minimum and maximum allele frequencies that did not significantly decrease the likelihood. Recursions and likelihood maximisation algorithms were written and compiled with Lazarus v1.0.10 (http://www.lazarus.freepascal.org/).

**Table 1:** *ace-1* phenotype diversity in Yamoussoukro and Yopougon.

		Resistance phenotype			
Locality	Year	[RR]	[SS] ([D_1_+])	[RS] ([D_1_+])	N
Yopougon	2012	0	40 (1)	15 (**3**)	55
	2015	1	13 (0)	45 (**25**)	59
	2016	1	20 (2)	37 (**18**)	58
Yamoussoukro	2012	0	21 (1)	25 (**14**)	46
	2015	1	12 (2)	35 (**15**)	48
	2016	0	9 (1)	30 (**15**)	39

Samples (identified by year and locality) originate from Assogba et al.’s (2018) study. [RR], [RS] and [SS] phenotypes were obtained through the “*ace-1* phenotype” PCR (see Material and Methods, Fig. 1B). The number of [D_1_+] individuals are indicated in brackets (“D_1_” PCR, Fig. 1B).

[RS, D_1_+] individuals are bolded, they are expected to carry at least one D_1_ allele.

#### Discriminating new D alleles from D_1_ allele and standard heterozygotes

**Fig. 2:**
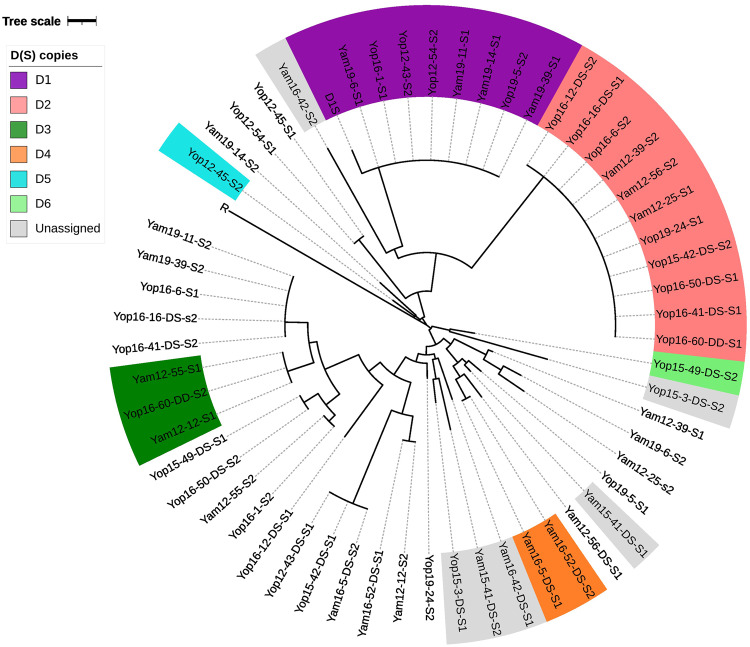
Diversity of the *ace-1* S and D(S) sequences in individuals displaying triple peaks. This phylogram represents the diversity of the S copies retrieved (TA cloning/Sanger sequencing and/or Nanopore sequencing, see Supp. Info. Table 2) from individuals displaying triple peaks in the mix sequence of the “*ace-1* resistance phenotype” PCR product (see Materials). Samples are coded as follows: locality (Yam for Yamoussoukro and Yop for Yopougon)/sampling year and individual number (-x). The 12 samples whose genotype was obtained through short-read sequencing are further coded with DS (duplicated heterozygote) or DD (duplicated homozygote). For each individual, the two S copies are indicated as copy S1 and copy S2 (assigned randomly). Sequences identified as single-copy S alleles are not highlighted. Copies identified as probable D(S) copies (see text) are highlighted according to the corresponding putative D allele (legend). Unassigned sequences, i.e. S sequences that are found in an individual carrying a D allele, but that cannot yet be assigned to S or D(S) for lack of data, are highlighted in grey. NB: the haplotypes assigned to D_1_(S), including the D_1_ controls, differed by one mutation from the canonical sequence (GenBank: KM875635.1).

Before this study a single D allele had been characterised in *An. gambiae s.l*., referred to as D_1_ (Assogba et al. 2018; Grau-Bové et al. 2021). For each population and year, we tested whether this allele alone could explain the estimated frequency of D alleles, or if more alleles (hereafter D_i_) could segregate in the populations. To do so, we used a PCR-RFLP test specific to the D_1_ susceptible *ace-1* copy (“D_1_” PCR-RFLP-test, Supp. Info. Table 1; Assogba et al. 2015) on all individuals with an [RS] phenotype (those that could harbour a D allele): the same 817 bp fragment of the *ace-1* gene is PCR-amplified and an *AvaI* restriction site specific to the D_1_(S) copy distinguishes between D_1_ carriers ([D_1_+] phenotype) and individuals that do not carry D_1_ ([D_1_-] phenotype; Fig. 1B-2). We then compared a model considering only three alleles (R, S, D_1_) with models considering either four (R, S, S_D1_, D_1_) or five alleles (R, S, S_D1_, D_1_, D_i_), using likelihood ratio tests (Labbé et al. 2009; Milesi et al. 2016); we took into consideration the possibility of occurrence of single-copy susceptible alleles S_D1_, carrying the same *AvaI*-diagnostic mutation as the D_1_(S) copy (Table 2).

**Table 2:** Estimated allele frequencies under different models.

A						
Model A- 3 alleles model						
Locality	Year	*R*	*S*	*D*	LRT (*χ*^2^)	*p-value*
**Yopougon**	**2012**	0.00 [0.00:0.18]	0.85	0.15 [0.00:0.22]	2.38	0.12^NS^
	**2015**	0.13 [0.03:0.27]	0.47	0.40 [0.23:0.55]	24.42	7.73 × 10^−7^***
	**2016**	0.13 [0.03:0.27]	0.61	0.27 [0.11:0.41]	11.94	5.50 × 10^−4^***
**Yamoussoukro**	**2012**	0.00 [0.00:0.20]	0.68	0.32 [0.10:0.43]	9.27	2.33 × 10^−3^**
	**2015**	0.14 [0.03:0.29]	0.53	0.33 [0.16:0.49]	14.84	1.17 × 10^−4^***
	**2016**	0.00 [0.00:0.22]	0.5	0.50 [0.26:0.63]	19.27	1.13 × 10^−4^***

B					
Model B- 4 alleles model					
Locality	Year	*R*	*S*	*S*_*D1*_	*D*_*1*_
**Yopougon**	**2012**	0.11 [0.06:0.18]	0.85	0.01 [0.00:0.05]	0.03 [0.01:0.07]
	**2015**	0.25 [0.16:0.34]	0.51	0.00 [0.00:0.03]	0.24 [0.16:0.33]
	**2016**	0.21 [0.14:0.30]	0.6	0.03 [0.00:0.08]	0.16 [0.09:0.24]
**Yamoussoukro**	**2012**	0.14 [0.08:0.23]	0.68	0.02 [0.00:0.07]	0.16 [0.09:0.25]
	**2015**	0.27 [0.18:0.38]	0.54	0.04 [0.01:0.11]	0.15 [0.08:0.24]
	**2016**	0.24 [0.15:0.36]	0.53	0.02 [0.00:0.10]	0.20 [0.11:0.31]

C								
Model C- 5 alleles model								
Locality	Year	*R*	*S*	*S*_*D1*_	*D*_*1*_	*D*_*i*_	LRT (*χ*^2^)	*p-value*
**Yopougon**	**2012**	0.00 [0.00:0.17]	0.84	0.01 [0.00:0.05]	0.03 [0.00:0.07]	0.12 [0.00:0.20]	1.6	0.21^NS^
	**2015**	0.13 [0.03:0.27]	0.47	0.00 [0.00:0.04]	0.24 [0.16:0.33]	0.16 [0.01:0.31]	4.61	0.03*
	**2016**	0.13 [0.03:0.27]	0.58	0.03 [0.00:0.09]	0.16 [0.09:0.24]	0.11 [0.00:0.25]	2.37	0.12^NS^
**Yamoussoukro**	**2012**	0.00 [0.00:0.20]	0.67	0.02 [0.00:0.07]	0.16 [0.09:0.25]	0.16 [0.00:0.26]	2.3	0.13^NS^
	**2015**	0.14 [0.03:0.29]	0.49	0.04 [0.01:0.12]	0.15 [0.08:0.24]	0.18 [0.02:0.35]	4.99	0.03*
	**2016**	0.00 [0.00:0.22]	0.47	0.03 [0.00:0.11]	0.20 [0.11:0.31]	0.30 [0.07:0.44]	7.07	0.01**

For each locality and sampling year, the frequencies of the different alleles (along with their support limits, i.e. roughly equivalent to 95% confidence intervals, brackets) have been estimated using a maximum likelihood approach under different assumptions (see text).

**Fig. 3:**
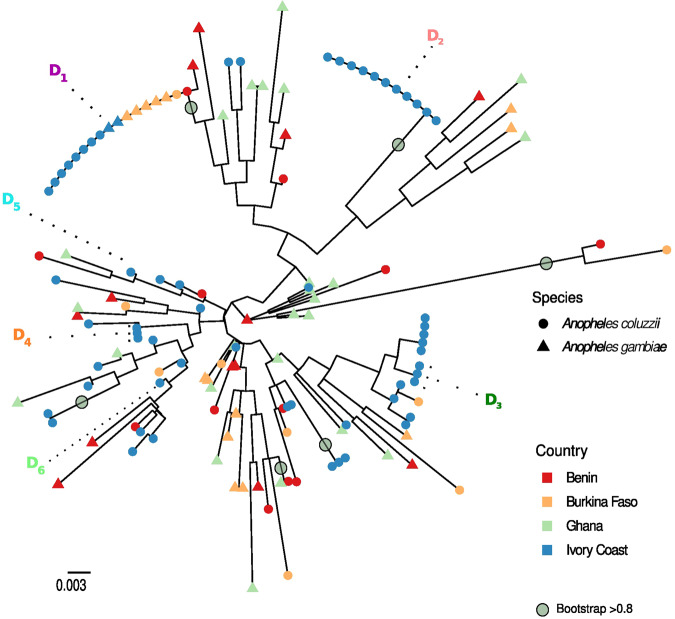
*ace-1* S diversity in West Africa. Maximum likelihood tree of *A. coluzzii* and *A. gambiae* S sequences from West Africa. For each sequence, species (triangle for *A. gambiae* and circle for *A. coluzzii*) and geographical origin (Benin in red, Burkina Faso in orange, Ghana in green, and Ivory Coast in blue, as in the inlet map) are indicated. Accession numbers can be found in Supp. Info. Table 3. The D(S) copies identified in the present study are also indicated, as well as the bootstraps confidence ≥0.8 with grey circles.

Model A - The first model considers only the phenotypes resulting from the “*ace-1* phenotype” test, thus three alleles, R, S and D, for three phenotypes and six genotypes (Fig. 1B). LRT corresponds to the likelihood ratio test between this model and another one without any D allele (chi-square distribution with 1 degree of freedom). Model B **-** The second model considers the phenotypes resulting from the combination of the “D_1_” and “*ace-1* phenotype” tests, i.e. adding the specific information on D_1_ frequency. Four alleles are thus considered, R, S, S_D1_ (as some [SS] are also [D_1_+]) and D_1_, for five phenotypes and nine genotypes (Fig. 1B-3, without genotypes including the D_i_ allele). Model C- The third model analyses the same data, but considers five alleles, R, S, S_D1_, D_1_ and D_i_ (i.e. at least another D allele), for 5 phenotypes and 13 genotypes (Fig. 1B-3, all genotypes). The *p*-value of the LRT comparing models B and C^a^ (chi-square distribution with 1 degree of freedom) is indicated for each population (if significant, model C better fits the data than model B).

^a^Models A and B cannot be compared using LRT as they are not fitted to the same dataset (3 vs. 5 phenotypes).

The significance of each *p-value* is indicated as follows: *NS* Non Significant, *p-*value* > 0.05, **p-*value* > 0.01, ***p-*value* > 0.001.

Our goal was then to characterise the *ace-1* haplotypes present in these potentially new D_i_ alleles. We first sequenced (Sanger sequencing ABI 3500 xL, Applied Biosystems by Thermo Fisher Scientific) the 817 bp *ace-1* PCR product for all the [RS D_1_-] (Fig. 1B-3), i.e. individuals that could carry a D but not D_1_ (except for three controls). If, as expected, these individuals harbour at least one D_i_ allele (genotypes D_i_R, D_i_S or D_i_D_j_), then up to three *ace-1* haplotypes should be present in the PCR product (D_i_(R), D_i_(S) and another one), which would result in SNPs with multiple peaks in the Sanger sequence (e.g. two peaks for an heterozygote). Providing the different haplotypes carry different SNPs, one could expect diagnostic “triple peaks” (positions at which three different SNPs can be found) in this mixed sequence. This enables discrimination of D_i_ carriers from standard RS heterozygotes. Although powerful to detect new D alleles, this approach can lead to an underestimation of the new D_i_ allele frequencies: when the *ace-1* haplotypes are similar between D alleles and single-copy resistance or susceptible alleles, triple peaks would not be detected.

### Heterogeneous duplication diversity

To identify the different haplotypes present in the *ace-1* PCR products of D_i_ carriers (i.e. “triple-peak” individuals), we used two approaches: (i) Sanger sequencing, which requires a preliminary TA cloning step to provide individual haplotypes from the mixed products, and (ii) an approach initially developed to assess the diversity of *Wolbachia cid* genes multigenic family, which is less tedious and more sensitive than TA cloning (Namias et al. 2023). In this approach, the PCR product is directly sequenced using Nanopore long-reads: each read then corresponds to one individual haplotype.

#### TA cloning/sanger sequencing

We purified the *ace-1* PCR products of 22 “triple-peak” individuals, using the BS664-250 Preps EZ-10 Spin Column PCR Purification kit (New England BioLabs, Evry France). The purified products were then cloned (TOPO TA Cloning Kit pCR 2.1-TOPO Vector and TOP10F’ invitrogen bacteria). For each individual, we genotyped 24 clones (Supp. Info. Table 2) using the *AluI* RFLP test (to discriminate R and S copies). All R haplotypes recovered so far in *An. gambiae s.l*. were strictly identical on this 817 bp fragment: we Sanger-sequenced one resistance clone (i.e. D(R) or R), and at least 11 susceptible clones (i.e. D(S) or S; ABI 3500 xL, Applied Biosystems by Thermo Fisher Scientific).

#### Nanopore sequencing of ace-1 PCR products

We directly sequenced the purified ace-1 PCR product of 12 “triple-peak” individuals (with a mean ≈1000X coverage for each individual) using Nanopore long-reads technology to capture, in a single read, each full 817 bp amplicon. Six of these individuals, which had been previously analysed with the TA cloning/Sanger sequencing approach, served as controls to assess the reliability of the Nanopore sequencing-based approach (Supp. Info. Table 2). We then adapted the bioinformatic pipeline developed by Namias et al. (2023): reads were mapped on a reference file containing two reference haplotypes, one R and one S, using *minimap2 v.2.24* (Li 2018) with the options *map-ont* and without secondary alignments. SNPs were then called using *bcftools 1.15*, with the *config*-*ont* option, and a minimum mapping quality of 10. Finally, haplotype phasing was performed using *WhatsHap 1.4* (Martin et al. 2023) with the default options. As mentioned in Namias et al. (2023), some heterozygous SNPs on the S haplotypes were not called: although they were supported by a high number of reads, the read distribution did not fit with a diploid framework (with only two S copies). We used the script provided by Namias et al. (2023) to recover those SNPs.

#### *ace-1* haplotype trees

To assess the diversity of *ace-1* haplotypes obtained through the two approaches, we aligned and compared them using a Neighbour-Joining phylogram compiled by *MEGA* software (MEGA11: Molecular Evolutionary Genetics Analysis version 11; Tamura et al. 2021). We added several known *ace-1* haplotypes to this phylogram: an R reference haplotype and the reference D_1_(S) copy haplotype. We used the information from molecular tests, whole genome sequencing (see below), *ace-1* PCR product long-read sequencing and haplotype frequency to discriminate the haplotypes corresponding to a D(S) copy from those corresponding to a single-copy S allele, in each individual.

To infer the geographical origin of the newly characterised D alleles, we then added publicly-available *ace-1* S haplotypes to our alignment, from samples collected in the same time period as or samples and in Ivory Coast or nearby countries (Ghana, sequences from Weetman et al. 2015; and Benin, Burkina Faso and Ivory Coast from Assogba et al. 2018; see Supp. Info. Table 3). We then computed a maximum likelihood phylogram using a Tamura 3 parameters model with gamma distributed invariant site (G + I) mutation rates (best model fit determined with MEGA11). The phylogram was then plotted using the *ggplot2* (Wickham et al. 2016) and *ggtree* packages (Xu et al. 2022) using the R software (v.4.2.2, R Core Team 2022; https://www.R-project.org/).

### Duplication architecture

#### Genomic characterisation

To determine the structural architecture of the newly characterised D_i_ alleles, we followed the protocol developed by Assogba et al. (2016) for D_1_. The whole genomes of twelve “triple-peak” individuals were sequenced using Illumina paired-end sequencing (WGS, 150 bp reads, 350 bp insert-size; Supp. Info. Table 2). Reads from each individual were mapped to the *An*. *gambiae* PEST reference genome assembly (AgamP4.13; https://www.vectorbase.org) using the *bwa* (*-mem*) algorithm (Li and Durbin 2009). The per-base depth of coverage (*pb*DoC) between positions 3,436,000 and 3,639,000 of the 2R chromosome (*ace-1* lies between positions 3,484,107 and 3,495,790) was obtained using the *samtools* suite (Danecek et al. 2021). We then standardised them (*pb*DoC_*std*_), dividing each by the average *µ*DoC calculated over the whole 2R chromosome (*pb*DoC_*std*_ = *pb*DoC/*µ*DoC) and plotting the *pb*DoC_*std*_ along this chromosome, using R software. It allowed a fine scale observation of the structure of duplications (location, size, gene copy-number, etc.). To determine the precise position of the duplication breakpoints, we isolated reads mapping at ±1 kb from the putative breakpoints determined from the *pb*DoC_*std*_ graphs and analysed the insert-size among discordant paired-reads (i.e. paired-reads from each side of the junction between amplicons would map on each extremity of the amplicons, with an apparent insert-size equal to the amplicon size) and the frequency of soft-clipped reads (i.e. the reads encompassing the junction and mapping partially on each extremity of the amplicons; see Assogba et al. 2016, Fig. S1).

#### Molecular validation of structural homologies

Assogba et al. (2016) developed a diagnostic PCR test for R^x^ and D_1_ duplications, which amplifies a 460 bp sequence overlapping the junction between the amplicons (Supp. Info. Table 1, “Junction”). We used this PCR to further assess whether the newly identified D_i_ alleles also shared the junction and the breakpoints of both D_1_ and R^x^ alleles, using susceptible [SS] individuals as controls.

## Results

### Ivory Coast populations are highly polymorphic for D alleles

We first analysed samples collected in two populations of Ivory Coast (Yamoussoukro and Yopougon) for which resistance was monitored between 2012 and 2016 (Assogba et al. 2018) (Table 1). We built the first model (Model A) to analyse the “*ace-1* phenotype” data and found that the two populations showed a significant excess of heterozygotes compared to panmixia, suggesting the presence of D alleles at relatively high frequencies (>0.15; Table 2A). Using the specific “D_1_” molecular test based on a single diagnostic mutation (Assogba et al. 2015), we built a second model (Model B) taking this information into account (Fig. 1B-3), including the rare S allele, subsequently named S_D1_ and identified from [SS] individuals positive for this “D_1_” test (Table 1). It showed that D_1_ alone did not explain the observations as well as the third model (Model C) which considered several D alleles (model C fitted significantly better than model B for three out of the six populations, Yopougon 2015, and Yamoussoukro 2015 and 2016, *p* < 0.05, Table 2C), with more coherent R and overall D frequencies (Table 2A, Table 2C vs Table 2B). This strongly supports the presence of at least one other D allele segregating in these populations. Interestingly, D_1_ was not always the most frequent D allele (e.g. Yamoussoukro 2015 and 2016, Table 2C).

To identify the alleles present in these populations, we first Sanger-sequenced a fragment of the *ace-1* locus for RR individuals. Only one single resistance haplotype was found (already known from previous studies; Weill et al. 2003; Djogbénou et al. 2008; Assogba et al. 2016; Grau-Bové et al. 2021), i.e. no other R sequence-allele (although there were probably different copy-numbers-alleles; Assogba et al. 2016; Djogbénou et al. 2015).

The only other known resistance allele was D_1_. We amplified the same PCR fragment in [RS, D_1_-] individuals, (individuals that could carry a D but not D_1_; *N* = 97, Table 1), plus three D_1_ carriers as controls. The PCR product mixes several haplotypes, both R and S, which results in multiple peaks for SNPs in the Sanger sequence (Fig. 1A, see “Discriminating new D alleles from D_1_ allele and standard heterozygotes”, Materials and Methods). As expected, some individuals (N = 22) displayed several positions with triple peaks (henceforth “triple-peak” individuals), confirming the existence of at least three haplotypes (R and S) in the mix and the presence of other D alleles (D_i_) segregating in these *An. gambiae s.l*. populations. We found six more “triple-peak” individuals in new samples collected in 2019 in Yopougon and Yamoussoukro (among 27 [RS] individuals analysed for each population), consequently, a total of 28 “triple-peak” individuals from 2012 to 2019 were identified.

To describe the diversity of the D_i_ resistance alleles, we used two approaches. First, Sanger sequencing of PCR products, which required a preliminary TA cloning step to get individual R and S haplotypes from the mix. As this protocol is tedious and difficult to apply to large numbers of individuals, we also tested another approach, using Nanopore long-reads sequencing of PCR products to directly access the various haplotypes carried by each individual (one read corresponds to one haplotype; Namias et al. 2023). Over the 28 individuals “triple-peak”, 22 were cloned and 12 were Nanopore sequenced (Supp. Info. Table 2). Six individuals among the cloned 22 served as controls for the Nanopore approach (Namias et al. 2023): adapting the previously described pipeline, we were able to recover the exact same haplotypes with both approaches (the ≈300X high coverage of each haplotype allows easily correcting the PCR and/or sequencing errors). This demonstrates the robustness of Namias et al.’s (2023) approach, which could be used to process much larger samples in the future.

As expected, for each “triple-peak” individual (carrying at least one D allele), three different *ace-1* haplotypes were identified, one R (119S), and two S (119 G) with different SNPs. The R haplotypes of all individuals were identical to D_1_(R) (the R copy carried by D_1_) and to the haplotype recovered from RR individuals: as a result, a unique sequence carrying the G119S mutation is present in all resistance alleles, whether R or D. The different D resistance alleles differed only in the S copies they carried (or D(S)): we found 26 different S haplotypes, which could be a D(S) or a S single-copy allele (see Fig. 1B “combined PCR”). They differed by only a few mutations, mostly found in introns, resulting in a relatively low divergence (*d* = 0.012; *d*_*exons*_ = 0.008 *vs. d*_*introns*_ = 0.027). To discriminate the D(S) copies from the S alleles, we compiled a neighbour-joining phylogram with S haplotypes recovered from the 28 “triple-peak” individuals.

We then assigned the most likely haplotypes as follow:

- i) We expected the D(S) haplotypes to be more frequent as they are directly selected for in the presence of insecticides (they provide resistance) and our protocol selected specifically for D-carriers; therefore, clusters of identical S haplotypes were more likely to correspond to D(S) copies than to S alleles (Supp. Info. Fig. 1A). Moreover, when several individuals had a first S haplotype in a cluster, their second S haplotype would often be different and attributed to single-copy S alleles.
- ii) This approach was first validated by the observation of an expected cluster corresponding to D1(S) (purple, Fig. 2). The second haplotypes found in the same individuals were different, as expected. For example, the individuals Yam19-11 and Yam19-14 (Fig. 2, Supp. Info. Table 4): two haplotypes (Yam19-11-S1 and Yam19-14-S1) were similar to D_1_(S), but the two others (Yam19-11-S2 and Yam19-14-S2) were different; they were identified as D_1_S individuals.
- iii) Similarly, a second large cluster was found (pink, Fig. 2), which could unambiguously be assigned to a second D allele, henceforth, D_2_.
- iv) A third and a fourth smaller clusters were found, with respectively three and two individuals sharing one S haplotype. We tentatively named them D_3_(S) and D_4_(S) (dark green and orange, resp., Fig. 2).
- v) Rather than inflating the number of potential D alleles, we chose to conservatively consider individuals carrying one haplotype similar to D_1_(S) or D_2_(S) as DS heterozygotes, even if the second S haplotype was found in another cluster, so they could actually be D_i_D_j_ heterozygotes (e.g. Yam19-39 and Yam19-11 carried either D1(S) or D2(S), but their second S haplotypes clustered together; Fig. 2, Supp. Info. Table 4). This parsimonious approach was further supported by genomic analyses (see below) that confirmed three such individuals were DS heterozygotes (Yop16-41, Yop16-6, Yop16-16; Supp. Info. Fig. 2). Following this principle, we identified the D(S) copy of Yop12-45 as Yop12-45-S2, tentatively named D_5_(S) (light blue, Fig. 2), because Yop12-45-S1 was identical to Yop12-54-S1, while Yop12-54-S2 was identical to D1(S) (Fig. 2, Supp. Info. Table 4).
- vi) For some individuals, the total number of *ace-1* copies they carried was independently known from genomic analyses (WGS, see Material and methods, Supp. Info. Table 5): Yop16-60 carried four copies (Fig. 3), but only three different haplotypes, one R and two S. Yop16-60 carried two different D alleles, it was a D_2_D_3_ heterozygote (Fig. 2): Yop16-60-S1 was identical to D_2_(S), and Yop16-60-S2 belonged to the tentative D_3_(S) cluster, which incidentally confirmed the existence of the D_3_ allele.
- vii) Conversely, genomics showed that Yop16-50 carried three *ace-1* copies (Supp. Info. Fig. 2) and was a DS heterozygote. As Yop16-50-S1 was identical to D_2_(S), it strongly suggested that Yop16-50-S2 was the single-copy S allele. Yop15-49-S1, which was identical to Yop16-50-S2 (Fig. 2, Supp. Info. Table 4), was most probably an S allele too. Consequently, as Yop16-49 carried a duplicated allele (one R and two S haplotypes), it was the second S haplotype, Yop15-49-S2, that was the D(S) copy (despite being isolated in the tree; Fig. 2); this new allele has been named D_6_.
- viii) For the last three D-carriers, Yam15-41, Yam16-42 and Yop15-3, both S haplotypes have single occurrences in the tree (Fig. 2, Supp. Info. Table 4). Although each carried at least one new D allele, hence D_7_, D_8_ and D_9_, which S haplotypes corresponded to their D(S) copies remained undetermined.

From the 28 analysed triple-peak individuals (56 alleles in total), we were able to conservatively infer that at least nine different D sequence-alleles (including D_1_) were segregating in Yamoussoukro and Yopougon. From the same 28 individuals, we also recovered 17 susceptible S alleles (Fig. 2 and Supp. Info. Table 4). For comparison, only one other resistance haplotype, R, was found in our samples, the same as that encountered in across West Africa. Apart from D_1_, D_2_ and D_3_, their D(S) haplotype identification remained however unsure. Different D alleles (up to four) were recovered in each population and year (Supp. Info. Table 4), indicating that the two populations remained polymorphic for these resistance alleles from 2012 to 2019 (>70 generations). D_1_, D_2_ and D_3_ in particular, were found over the whole period, while the other D alleles were found only once. However, it does not mean that these alleles have appeared and disappeared rapidly: our study protocol was designed to assess the overall diversity of D alleles, but not to infer their frequency, or even their presence over time (the sampling size was too limited, one to eight individuals per year and population, and limited to those displaying the triple-peak signal).

Nine D sequence-alleles is a minimum estimate of the real diversity of the duplicated resistance alleles (our approach was not exhaustive). This relatively high diversity, segregating in only two Ivory Coast populations, immediately begs the question of their molecular origins: i) could a single original duplication event followed by secondary rearrangements (e.g. recombinations, deletions) have generated this diversity? or ii) are multiple independent events of duplication required, one for each allele? To try and answer this question, we used two approaches, the first one based on phylogeography, the second using genomics.

### *ace-1* haplotypes do not show structure at the geographical or species level

We first tried to assess whether the different D alleles could be associated with a particular geographical origin in West Africa. We added 27 *ace-1* S haplotypes (from 78 sequenced individuals) from neighbouring countries (Benin, Burkina Faso, Ghana) of both *An. gambiae* and *An. coluzzii* and computed a phylogenetic model (Fig. 3; Tamura 3 parameters G + I, see Materials). This revealed no clustering pertaining to geographical origin or species for the different S copies (whether S or D(S)). Despite testing different models of evolution, we found that close or identical S copies can be found in all countries, and that none of the different D(S) copies are associated with a particular country. In fact, the only highly supported nodes are those of the D(S) clusters (Fig. 3). The diversity is relatively low and mostly concentrated in the introns. Translating the exonic part of the *ace-1* haplotypes carried by D alleles showed that all mutations were synonymous, except G119S for the D(R) haplotypes.

Overall, we have no evidence for whether the different D alleles identified in the present study have originated in the populations where we found them or elsewhere, and cannot rule out the possibility of a unique origin for all.

### All D alleles share a common genomic architecture

We then took advantage of the bioinformatic approach developed by Assogba et al. (2016) to analyse the genomic architecture of these alleles (copy number, amplicon size, breaking points, etc.): we already knew that D_1_ and R^x^ alleles share the same breaking points, but what about these new D alleles? Different genomic structures would support independent origins.

#### Copy number and amplicon size

**Fig. 4:**
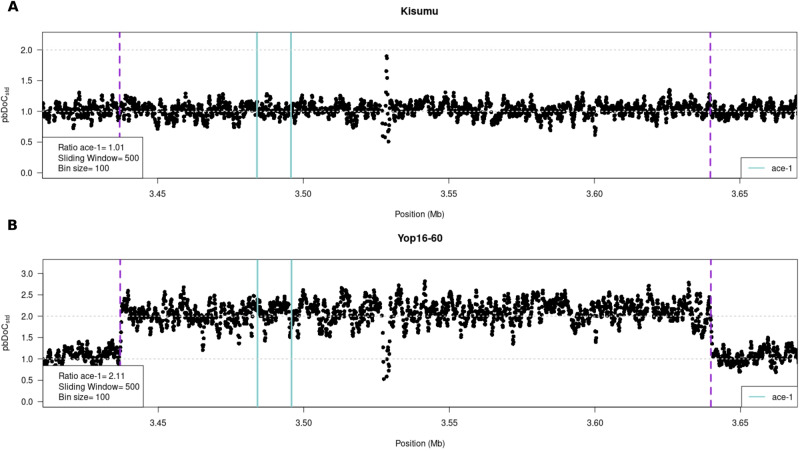
Genomic architecture of the *ace-1* D alleles. In each graph, we presented the variation of the standardised per-base depth of coverage (*pbDoC*, with 1 being the mean *pbDoC* calculated over the whole chromosome) along the region of interest, from 3.4 to 3.7 MB of chromosome 2R. Each dot is the mean *pbDoC* calculated every 100 bases (bin size) over 500-base sliding windows. The purple dashed lines represent the amplicon limits of the D and R alleles (Assogba et al. 2018); the cyan lines represent the *ace-1* gene location. **A** The susceptible strain Kisumu is the single-copy S allele reference, with no particular variation of *pbDoC* (mean = 1). **B** The second graph represents the *pbDoC* variation for the individual Yop16-60 (as a representative example of the D alleles analysed in the present study; similar graphs for the other individuals analysed are shown in Supp. Info. Fig. 1). A twofold increase reveals the amplicon size and location, similar to D; it is consistent with a D/D genotype (two S and two R copies). All D alleles share the same breakpoints as D; however, the other individuals display only a 1.5 increase, as expected for genotypes DS (two S and 1 R copies; Supp. Info. Fig. 1).

Twelve individuals carrying different D alleles (D_2_, D_3_, D_4_, D_7_, D_8_, D_9_) were sequenced using Illumina paired-end sequencing (Supp. Info. Tables 2 and 4; we could not get enough DNA for individuals carrying D_5_ and D_6_). The reads were first mapped onto the reference *A. gambiae* PEST genome (Vector-Base; AgamP4.13). We also mapped short-reads from the susceptible reference strain Kisumu as a non-duplicated reference. For each individual, we calculated a standardised depth of coverage (*pb*DoC_*std*_) for each base in a region surrounding *ace-1* (see Materials and Methods). As expected, Kisumu’s *pb*DoC_*std*_ remained close to 1 over the whole region (Fig. 4A). By contrast, all D-carriers displayed a consistent *pb*DoC_*std*_ increase over a 203 kb region encompassing the *ace-1* locus, similar to that seen for D_1_ (Fig. 4B and Supp. Info. Fig. 2). For 11 individuals we observed a 1.5-fold *pb*DoC_*std*_ increase (1.5 ± 0.14 for the duplicated region *vs*. 1 ± 0.16 for the flanking non-duplicated regions), which is consistent with a DS genotype (Supp. Info. Fig. 2). Yop16-60, displayed a 2-fold *pb*DoC_*std*_ increase in the same area (Fig. 4B), suggesting a DD genotype (actually D_2_D_3_, see D(S) copies identification above).

#### Breakpoint positions

We further combined information from insert-size among discordant read pairs, i.e. pairs overlapping the amplicon junction, and local enrichment in soft-clipped reads, i.e. reads overlapping the amplicon junction, to precisely map the breakpoints of the duplication. For all the analysed D alleles, (i) the insert-size of the discordant read pairs showed the duplication to be 203 kb, and (ii) a significant increase of soft-clipped reads was found on positions similar to the 5’ and 3’ breakpoint positions previously identified for D_1_ (position 3,436,927 and position 3,639,836; resp.; Fig. 3B; Assogba et al. 2016). We finally submitted these individuals to the specific PCR test designed by Assogba et al. (2016) that amplifies a 460 bp pb fragment overlapping the amplicon junction in D_1_ and R^x^ alleles. The fragment was amplified in all 12 individuals.

Together, this PCR test and the genomic analysis indicate that all the D alleles share the exact same genomic architecture, i.e. two amplicons only, with the same boundaries and sizes (without any internal deletion as seen in some R^x^ alleles, Assogba et al. 2016, 2018).

## Discussion

### An unsuspected duplicated allele diversity: beyond the spotlight effect

So far, in *An. gambiae s.l*., the only *ace-1* SNP that has been linked to OP/CX insecticide resistance is G119S (Weill et al. 2003; Djogbénou et al. 2008; Assogba et al. 2016; Grau-Bové et al. 2021; a couple of other substitutions have been found in other mosquito species e.g. Alout et al. 2007a, 2007b). Moreover, it appears that this mutation occurred only once in *An. gambiae s.l*. (introgressing from *An. coluzzii* to *An. gambiae s.s*.; Djogbénou et al. 2008), so that a single R haplotype has rapidly spread over all West Africa (Weill et al. 2003; Djogbénou et al. 2008; Assogba et al. 2016, this study). Interestingly, no single-copy R allele has been found, only homogeneous duplications, with various copy-number-alleles of repeated identical R haplotype (R^x^, Fig. 1A; Assogba et al. 2016; Grau-Bové et al. 2021).

Despite indications of more variation (Grau-Bové et al. 2021), the only other resistance allele known in *An. gambiae s.l*. was one heterogeneous duplication, D_1_, also found across all West Africa (Djogbénou et al. 2008, 2009; Assogba et al. 2016): this allele carries one R and one S copy, resulting in a [RS] phenotype using the usual molecular test (Fig. 1). Our study showed that significant excess of apparent heterozygotes in two populations of Ivory Coast could not be explained by the presence of D_1_ alone (Table 2). Through a multi-approach genotyping and sequencing protocol, we have further evidenced high diversity of *ace-1* resistance alleles in West African *An. gambiae s.l*., with eight new D sequence-alleles, found in only two Ivory Coast populations. All D alleles carry one identical R haplotype, but carry a unique S haplotype, i.e. their D(S) copy is different (similarly to most of the 27 different D alleles described so far in *Cx. pipiens*; Milesi et al. 2018). They also share the same genomic architecture as D_1_, *only* two *ace-1* copies, the same amplicon size and breakpoints. The D allele diversity is high compared to a unique R sequence-allele, but it is only half that of single-copy S alleles, segregating in the same populations (17 different S haplotypes, Supp. Info. Table 4).

The fact that only D_1_ had been described since the seminal work of Djogbénou et al. (2008), despite regular surveys (Djogbénou et al. 2008; Assogba et al. 2016, 2018; Grau-Bové et al. 2021; Kouamé et al. 2023), highlights a bias in the way these variants are studied: the “classical” approach, based on field-caught individuals, crossed in the laboratory with a reference susceptible strain (Labbé et al. 2007; Assogba et al. 2016; Milesi et al. 2018), will only retain genotypes frequent enough to be sampled, and also potentially important, individuals fit enough to survive and reproduce in the laboratory. It’s a major problem when studying duplicated alleles that are often coupled with strong deleterious effects (Innan and Kondrashov 2010; Schrider and Hahn 2010; Schrider et al. 2013; Milesi et al. 2018). On the other hand, surveys relying on specific molecular tests for a few diagnostic mutations are prone to a strong “spotlight effect”, i.e. they can only find what they are looking for, especially when these mutations are not directly causal for the resistance phenotype.

The last decade has seen a giant leap in sequencing and bioinformatics analyses based on NGS data, which are now affordable for extensive surveys of natural populations. However, there are also limitations when it comes to precisely describing (at the sequence level) structural variants in natural populations, as is the case in our study. For example, in Ivory Coast, Grau-Bové et al. (2021) suggested variation in the number of S copies in D alleles (i.e. copy-number-alleles) of which we found no evidence: all the D alleles identified in the present study carried only two copies, one S and one R haplotype. While we cannot exclude the existence of D copy-number-alleles, our analyses suggest that this discrepancy may come from how the number of copies were determined in these two studies. Identifying S/R copy-number ratio only from the ratio of allelic coverage at a single diagnostic position (here the G119S point mutation, see Grau-Bové et al. 2021) can lead to inaccurate copy-number estimations, especially with low depth of coverage (see simulations in Karunarathne et al. 2023). In our study, using the average depth of coverage across the whole *ace-1* gene to assess the number of copies and to deduce the genotype, proved to be more reliable (Supp. Info Table 5). Similarly, despite indications that suggested the potential existence of D sequence-alleles, Grau-Bové et al.’s study did not allow their specific identification, because haplotype reconstruction is particularly difficult from short-read data when several copies are present. We demonstrated the potential of long-read sequencing to overcome this issue. We described the same haplotypes through direct long-read sequencing of the PCR mix and bioinformatics analyses, and with the logistically heavy but reliable TA cloning/Sanger sequencing approach. As long-read sequences become more accessible and reliable, some limitations may reduce, especially for structural variant detection and study (Mantere et al. 2019; De Coster et al. 2021; Namias et al. 2023; although these methods are still limited for the amplicon sizes we are studying; see Hook and Timp 2023 for a review).

### The molecular origins of D alleles remain to be confirmed, although secondary recombinations are likely

The surprising diversity of D resistance alleles found in two populations poses questions on their origin(s) in *Anopheles* mosquitoes.

**Fig. 5:**
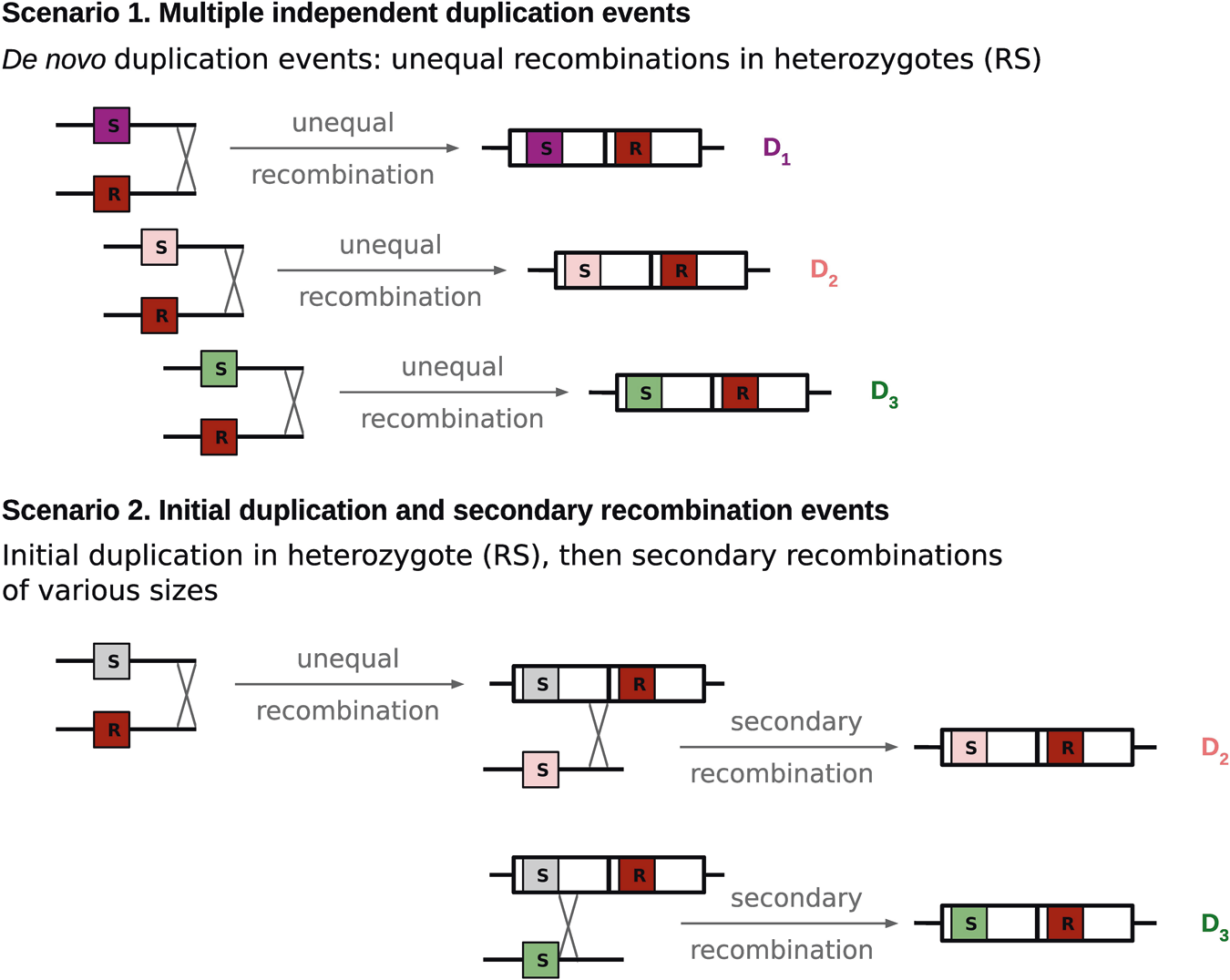
Possible scenarios for the origin of Ivory Coast *ace-1* duplications. Scenario 1 requires several independent duplication events on the same breakpoints, whereas scenario 2 considers a first duplication event followed by secondary recombinations occurring in the amplicon that bears the S copy (either the whole amplicon, or only the part containing the *ace-1* locus, or any size between). *NB: the alleles presented here are for illustration only, as the present study did not allow firmly distinguishing the two scenarios, or any secondary recombination span. Similarly, the oblique lines are used to illustrate the recombination events, but not a particular molecular mechanism*.

Deletions/duplications are frequent for multi-copy genes (e.g. esterases, Milesi et al. 2016), due to unequal recombination, so that the existence of different R^x^ copy-number-alleles is expected (note that all the copies have the same size exactly, although a secondary internal deletion has been described; Assogba et al. 2016, 2018). The existence of several D alleles carrying one identical R haplotype and different S haplotypes could be explained by two different scenarios, as proposed for D alleles in *Cx. pipiens* (Labbé et al. 2007; Milesi et al. 2018). The first one requires multiple independent unequal recombination events in RS heterozygotes (scenario 1, Fig. 5). The second scenario (scenario 2, Fig. 5) only requires one unequal recombination event, followed by secondary recombination event between the D(S) copy bound in the duplication (or one R copy of a R^2^ allele) and a single-copy S allele in heterozygous DS (or R^2^S) individual. These secondary recombination events could be limited to the *ace-1* sequence or much larger (up to 203 kb, i.e. encompassing all the genes embedded in the duplication). Note that D alleles could also be produced by reversion of a R copy, i.e. the reversion of the resistance mutation G119S to S119G; but the observed divergence between the S copies and the single R haplotype makes it unlikely here (Fig. 2).

In *Cx. pipiens*, both scenarios are probably at play: both D(R) and D(S) copies differ between some D alleles, which is expected in scenario 1; however, many share the same D(R) copy, and the D(S) copies are found as single-copy S alleles in the same populations, a pattern expected in scenario 2 (Milesi et al. 2018). In *An. gambiae*, all D alleles (as well as R^x^) display a strict structural homology, i.e. the exact same boundaries and breakpoints, which would require frequent and precisely localised de novo unequal recombination under scenario 1. While Assogba et al. (2016) did find a *harbinger* transposable element on the 3’ end of the amplicon, it is not evident that it fits such recurrent recombination events in the same genomic area. Therefore, scenario 1 does not appear to be the most parsimonious. The diversity of D alleles we observed was more likely to be generated by secondary recombination events between S copies. Single-copy S alleles with close haplotype sequences are found in the same populations (Fig. 2), further supporting the hypothesis that the diversity of D alleles was likely due to secondary recombinations, but the lack of geographic structure tends to weaken it (Fig. 4). To confidently evaluate those hypotheses, complete haplotypes of the duplicated alleles are required. This could soon be possible, with the improvement of long-read sequencing (but for the amplicon size, see above).

Nonetheless, both scenarios imply a high recombination rate in this genomic region, as these alleles are fairly recent in terms of evolutionary time: OP and CX insecticides have only been used for 50–60 years to control *An. gambiae s.l*. population; resistance was first reported in the late 1990’s-early 2000’s (Elissa et al. 1994; N’Guessan et al. 2003); the first reports of a D allele are even more recent (Djogbénou et al. 2008).

### How is the *ace-1* resistance alleles diversity maintained in the populations?

Several studies in *An. gambiae* or *Cx. pipiens* have shown that various resistance alleles at the *ace-1* locus conferred different fitnesses (e.g. Assogba et al. 2015, 2016 in *An. gambiae*, Labbé et al. 2007, 2014, Milesi et al. 2018, 2022 in *Cx. pipiens*). For the homogenous duplicated alleles R^x^, a larger number of copies (e.g. 5 *vs* 3) confers both a higher resistance level and higher selective disadvantages, although it does not follow a linear pattern (Assogba et al. 2016 in *An. Gambiae*; Milesi et al. 2022 in *Cx. pipiens*). These alleles appear to be selected in areas exposed to intense selective pressure, the copy-number-allele diversity is potentially being maintained by small insecticide treatment fluctuations (Assogba et al. 2018). On the other hand, D_1_, the only heterogenous duplicated allele known before the present study in *An. gambiae*, has been shown to confer a phenotype similar to standard heterozygotes RS: it provides more resistance than S but less than R^x^ alleles, and less selective disadvantages than R^x^ but more than S (Assogba et al. 2015, 2016), as observed for D alleles in *Cx. pipiens* (Labbé et al. 2014). These alleles are selected for in areas where insecticide treatments are moderate or fluctuating (Assogba 2015; 2016; Lenormand et al. 1998; Labbé et al. 2007, 2014; Milesi et al. 2018, 2017).

In the present study, in all samples except those from Yopougon in 2012, the resistant heterozygous phenotype was more frequent than the susceptible phenotype, and the frequency of [RR] individuals was very low (maximum one individual per sample, Table 2). Considering the predicted allelic frequencies, the overall D frequency remained globally stable from 2012 to 2019, despite a slight increase in the total frequency of the resistance alleles (R + D, binomial test for differences in the estimated S allele frequencies between the different years, *p* = 5.5 × 10^−11^ and *p* = 2.4 × 10^−4^ for Yopougon and Yamoussoukro, respectively; Supp. Info. Fig. 3). As the response to change in selective pressure is usually rapid for resistance alleles (even seasonal; Lenormand et al. 1999; Milesi et al. 2016; Milesi et al. 2017), it suggests that the insecticide treatments did not change much over the period of the study. The higher frequency of D over R^x^ among resistance alleles and the persistence of S alleles at relatively high frequencies, further suggests that these populations were exposed to moderate (or fluctuating) treatment intensities.

We found nine D sequence-alleles that differ only by their D(S) copy and up to four of them have been co-segregating in the same population over at least 70 generations (Supp. Info. Table 4). At first glance, these alleles are not expected to confer different resistance levels. Previous studies in *Cx. pipiens* suggests that the intermediate resistance level displayed by D alleles depends entirely on the association of an R and an S copy (i.e. one carrying the G119S mutation and the other not), but not on the sequence of the R or S alleles captured in the duplication (Labbé et al. 2007, 2014; Milesi et al. 2018). While D alleles are clearly selected for over the S and R^x^ alleles in conditions of intermediate selective pressures, the D sequence-alleles found here are expected to be neutral in terms of selective advantage. A first parsimonious explanation would be that the polymorphism observed in D alleles reflects the rate at which new D alleles are generated, i.e. the duplication rate—genetic drift equilibrium considering scenario 1, or the recombination rate - genetic drift equilibrium in scenario 2. In such cases, the diversity of D alleles should be similar to that of the S alleles, and their frequency spectrum should also be similar. However, the diversity of the D alleles is lower than the neutral expectancy provided by the diversity of S alleles found in the exact same individuals: in the 28 “triple-peak” individuals (56 alleles in total), we identified nine different D haplotypes over 29 D sequences, but significantly more S haplotypes (17 different haplotypes over 27 S sequences; binomial test, *χ*^*2*^ = 4.5, *df* = 1, *p* = 0.03). Similarly, some D alleles are found more often than the S alleles (Table 4 and Supp. Info. Fig. 1B): for instance, D_2_ and D_1_ were found 11 and 8 times, respectively (relative frequency over all D alleles = 0.38 and 0.28, resp.; Supp. Info. Fig. 1B), while one S allele was found five times and the second most frequent S three times (relative frequency over all S alleles = 0.18 and 0.11, resp.; Supp. Info. Fig. 1B). Our limited sample size and the fact that we captured one D_i_D_j_ heterozygote further suggests that the D alleles captured in this study may be quite frequent in natural populations (Yam 16–60 is a D_2_D_3_ heterozygote found among the five individuals genotyped in this year and population, Supp. Info. Table 4; its expected frequency under panmixia is *f*_*D2D3*_ = 2*f*_*D2*_*f*_*D3*_). It is unlikely that the D(S) copies would be a random sample of the S diversity, considering that our discovery approach was meant to maximise the diversity in D alleles while not affecting S allele diversity.

It is conceivable that the observed allelic diversity and frequencies of D alleles is influenced by both neutral processes (i.e. reflecting the recombination rate between D and S alleles), and non-neutral processes explaining their maintenance and increase in frequency. How could selection contribute to explaining the persistence of D resistance allele polymorphism at the population scale? In *Cx. pipiens* several populations are polymorphic for D alleles (Milesi et al 2018). These D alleles are associated with various deleterious effects expressed only in homozygotes and independent from *ace-1* (Labbé et al. 2014) and they complement each other (each D allele compensates for each other’s flaws, so that a D_i_D_j_ heterozygote is fine; Labbé et al. 2007; Milesi et al. 2018). Simulations have shown that such alleles can be maintained in the same population by frequency-dependent selection (Milesi et al. 2018). Gene duplications are extensive structural rearrangements that are known to be largely detrimental (Schrider et al. 2013; Katju and Berthogsson 2013), whether for structural reasons (e.g. gene disruption, deleterious mutation hitchhiking) or in relation to gene-dosage imbalance. It would not be surprising that similarly detrimental D alleles, that could complement each other, might be found in *An. gambiae s.l*. too. However, D_1_ was not sublethal when homozygous in lab experiments (although less fit than S in absence of insecticides; Assogba et al. 2015) and this frequency-dependent selection among D alleles should last only until S and R^x^ alleles are eliminated. On the other hand, S allele frequency remained relatively high and globally stable over the whole period of the study (Table 2, Supp. Info. Table 4), arguing for a complex balancing selection situation, as was observed in *Cx. pipiens* (Milesi et al. 2018). A definitive approach to assess the primacy of selection over neutral processes in the observed D diversity would require measuring the fitness of these new D alleles, either by establishing a mosquito line for each of these alleles, introgressed on a unique genomic background (e.g., Labbé et al. 2007; Assogba et al. 2015, 2016; Milesi et al. 2018) or by monitoring their dynamics in the populations over many years (e.g., Labbe et al. 2009; Milesi et al. 2016). Both require extensive long-lasting effort. By revealing the existence of this unexpectedly high polymorphism of the D allele in *An. gambiae s.l*. our study represents the first step in that direction.

## Conclusion

Altogether our findings highlight the relatively high local diversity and frequency of *ace-1* heterogeneous duplications implicated in the adaptation to OP/CX insecticides in *Anopheles* mosquitoes, particularly when compared to the regional uniqueness of the other resistance allele (R). This diversity likely results from frequent secondary recombination events between single-copy and duplicated alleles, not only restricted to *ace-1* but potentially involving a large portion of the 203 kb amplicon. Our study supports a role for selection in the maintenance of D allele polymorphism in the populations, but further investigations are required to better assess the relative roles of neutral processes and selection.

More generally, our study highlights the challenges that must be overcome when analysing large-scale structural variants (SV). The last 15 years have seen an increase of interest in the role of SVs in the adaptation process (e.g. Wellenreuther et al. 2019). Gene copy-number variations are a perfect example of genomic mutations that are particularly arduous to investigate for technical reasons (they are mostly just more of the same sequence, all mapping together on reference genomes), but also because of their often multi-allelic nature. For large-scale segmental duplications, our study of *An. gambiae s.l*. clearly demonstrates that both the number of copies (R^x^ alleles) and the specific sequence carried by the copies (D alleles) are relevant to understand their evolution. It also showed that the study of copy-number variations (CNVs) is prone to misinterpretations with rushed approaches (as both the number and the nature of the copies can affect the phenotype).

These large genomic mutations are frequent and ubiquitous (Emerson et al. 2008; Itsara et al. 2009; Reams et al. 2010; Langley et al. 2012; Katju and Bergthorsson 2013; Schrider et al. 2013; Remnant et al. 2013; Mérot et al. 2020), they played a decisive role in the evolution of living organisms and are still determinant in the adaptation process, even at the micro-evolutionary scale (e.g. Kondrashov 2012 and references therein); therefore they are worth the painstaking endeavour to study them.

### Supplementary information

Supplementary figures

Supplementary tables

## Data Availability

Sequences have been archived in Genebank. Accession numbers PRJNA971118, KP165332.1 to KP165342.1, KP165361.1, KP165362.1, and KP165373.1 to KP165384.1.

## References

[CR1] Alout H, Berthomieu A, Berticat CC (2007). Different amino-acid substitutions confer insecticide resistance through acetylcholinesterase 1 insensitivity in *Culex vishnui* and *Culex tritaeniorhynchus* (Diptera: Culicidae) mosquitoes from China. J Med Entomol.

[CR2] Alout H, Berthomieu A, Hadjivassilis A, Weill M (2007). A new amino-acid substitution in acetylcholinesterase 1 confers insecticide resistance to *Culex pipiens* mosquitoes from Cyprus. Insect Biochem Mol Biol.

[CR3] Alout H, Djogbénou L, Berticat C, Chandre F, Weill M (2008). Comparison of *Anopheles gambiae* and *Culex pipiens* acetycholinesterase 1 biochemical properties. Comp Biochem Physiol B Biochem Mol Biol.

[CR4] Assogba, Djogbénou BS, Milesi LS, Berthomieu P, Perez A, Ayala J (2015). An *ace-1* gene duplication resorbs the fitness cost associated with resistance in *Anopheles gambiae*, the main malaria mosquito. Sci Rep.

[CR5] Assogba BS, Milesi P, Djogbénou LS, Berthomieu A, Makoundou P, Baba-Moussa LS (2016). The *ace-1* locus is amplified in all resistant *Anopheles gambiae* mosquitoes: fitness consequences of homogeneous and heterogeneous duplications. PLoS Biology.

[CR6] Assogba BS, Alout H, Koffi A, Penetier C, Djogbénou LS, Makoundou P (2018). Adaptive deletion in resistance gene duplications in the malaria vector *Anopheles gambiae*. Evol Appl.

[CR7] Bourguet D, Roig A, Toutant J-P, Arpagaus M (1997). Aanalysis of molecular forms and pharmacological properties of acetylcholinesterase in several mosquito species. Neurochem Int.

[CR8] Collins FH, Mendez MA, Rasmussen MO, Mehaffey PC, Besansky NJ, Finnerty V (1987). A ribosomal RNA gene probe differentiates member species of the *Anopheles gambiae* complex. Am J Trop Med Hyg.

[CR9] Danecek, Bonfield P, Liddle JK, Marshall J, Ohan J, Pollard V (2021). Twelve years of SAMtools and BCFtools. Gigascience.

[CR10] David, J-P, Coissac, E, Melodelima, C, Poupardin, R, Riaz, MA, Chandor-Proust, A, & Reynaud, S (2010). Transcriptome response to pollutants and insecticides in the dengue vector Aedes aegypti using next-generation sequencing technology. BMC Genom, 10.1186/1471-2164-11-216 10.1186/1471-2164-11-216 PMC2867825 20356352

[CR11] De Coster W, Weissensteiner MH, Sedlazeck FJ (2021). Towards population-scale long-read sequencing. Nat Rev Genet.

[CR12] Devonshire A, Sawicki R (1979). Insecticide-resistant *Myzus persicae* as an example of evolution by gene duplication. Nature.

[CR13] Djogbénou L, Chandre F, Berthomieu A, Dabiré RK, Koffi A, Alout H, Weill M (2008). Evidence of introgression of the *ace-1*^*R*^ mutation and of the *ace-1* duplication in west African *Anopheles gambiae s. s*. PLoS ONE.

[CR14] Djogbénou LS, Assogba B, Essandoh J, Constant EAV, Makoutodé M, Akogbéto M (2015). Estimation of allele-specific *Ace-1* duplication in insecticide-resistant *Anopheles* mosquitoes from West Africa. Malar J.

[CR15] Elissa N, Mouchet J, Rivière F, Meunier JY, Yao K (1994). Sensibilité d’*Anopheles gambiae* aux insecticides en Côte d’Ivoire. Cahiers Santé.

[CR16] Emerson JJ, Cardoso-Moreira M, Borevitz JO, Long M (2008). Natural selection shapes genome-wide patterns of copy-number polymorphism in *Drosophila melanogaster*. Science.

[CR17] Favia G, Della Torre A, Bagayoko M, Lanfrancotti A, Sagnon N, Touré YT, Coluzzi M (1997). Molecular identification of sympatric chromosomal forms of *Anopheles gambiae* and further evidence of their reproductive isolation. Insect Mol Biol.

[CR18] Grau-Bové X, Lucas E, Pipini D, Rippon E, van ‘t Hof AE, Constant E, Dadzie S (2021). Resistance to pirimiphos-methyl in West African Anopheles is spreading via duplication and introgression of the *Ace1* locus. PLoS Genet.

[CR19] Guillemaud T, Lenormand T, Bourguet D, Chevillon C, Pasteur N, Raymond M (1998). Evolution of resistance in *Culex pipiens*: allele replacement and changing environment. Evolution.

[CR20] Hendry AP, Gotanda KM, Svensson EI (2017). Human influences on evolution, and the ecological and societal consequences. Philos Trans R Soc B Biol Sci.

[CR21] Hook PW, Timp W (2023). Beyond assembly: the increasing flexibility of single-molecule sequencing technology. Nat Rev Genet.

[CR22] Huchard E, Martinez M, Alout H, Douzery EJP, Lutfalla G, Berthomieu A (2006). Acetylcholinesterase genes within the Diptera: takeover and loss in true flies. Proc R Soc B Biol Sci.

[CR23] Innan H, Kondrashov F (2010). The evolution of gene duplications: classifying and distinguishing between models. Nat Rev Genet.

[CR24] Itsara A, Cooper GM, Baker C, Girirajan S, Li J, Absher D (2009). Population analysis of large copy number variants and hotspots of human genetic disease. Am J Hum Genet.

[CR25] Karunarathne P, Zhou Q, Schliep K, Milesi P (2023). A comprehensive framework for detecting copy number variants from single nucleotide polymorphism data: ‘rCNV’, a versatile r package for paralogue and CNV detection. Mol Ecol Resour.

[CR26] Katju V, Bergthorsson U (2013). Copy-number changes in evolution: rates, fitness effects and adaptive significance. Front Genet.

[CR27] Kondrashov FA (2012). Gene duplication as a mechanism of genomic adaptation to a changing environment. Proc R Soc B Biol Sci.

[CR28] Kouamé RMA, Lynd A, Kouamé JKI, Vavassori L, Abo K, Donnelly MJ (2023). Widespread occurrence of copy number variants and fixation of pyrethroid target site resistance in *Anopheles gambiae* (*s.l*.) from southern Côte d’Ivoire. Curr Res Parasitol Vector-Borne Dis.

[CR29] Kwon DH, Clark JM, Lee SH, Clarkt JM, Lee SH (2010). Extensive gene duplication of acetylcholinesterase associated with organophosphate resistance in the two-spotted spider mite. Insect Mol Biol.

[CR30] Labbé P, Berthomieu A, Berticat C, Alout H, Raymond M, Lenormand T, Weill M (2007). Independent duplications of the acetylcholinesterase gene conferring insecticide resistance in the mosquito *Culex pipiens*. Mol Biol Evol.

[CR31] Labbé P, Sidos N, Raymond M, Lenormand T (2009). Resistance gene replacement in the mosquito *Culex pipiens*: fitness estimation from long-term cline series. Genetics.

[CR32] Labbé P, Milesi P, Yébakima A, Pasteur N, Weill M, Lenormand T (2014). Gene-dosage effects on fitness in recent adaptive duplications: *ace-1* in the mosquito *Culex pipiens*. Evolution.

[CR33] Langley CH, Stevens K, Cardeno C, Lee YCG, Schrider DR, Pool JE (2012). Genomic variation in natural populations of Drosophila melanogaster. Genetics.

[CR34] Leister D (2004). Tandem and segmental gene duplication and recombination in the evolution of plant disease resistance genes. Trends Genet.

[CR35] Lenormand T, Harmand N, Gallet R (2018). Cost of resistance: an unreasonably expensive concept. Rethinking Ecol.

[CR36] Lenormand T, Guillemaud T, Bourguet D, Raymond M (1998). Appearance and sweep of a gene duplication: adaptive response and potential for new functions in the mosquito *Culex pipiens*. Evolution.

[CR37] Lenormand T, Bourguet D, Guillemaud T, Raymond M (1999). Tracking the evolution of insecticide resistance in the mosquito *Culex pipiens*. Nature.

[CR38] Li H, Durbin R (2009). Fast and accurate short read alignment with Burrows-Wheeler transform. Bioinformatics.

[CR39] Li H (2018). Minimap2: Pairwise alignment for nucleotide sequences. Bioinformatics.

[CR40] Mantere T, Kersten S, Hoischen A (2019). Long-read sequencing emerging in medical genetics. Front Genet.

[CR41] Martin, M, Ebert, P, Marschall, T (2023). Read-Based Phasing and Analysis of Phased Variants with WhatsHap. In: Peters, BA, Drmanac, R (eds) Haplotyping. Methods in molecular biology, vol 2590. Humana, New York, NY. 10.1007/978-1-0716-2819-5_8 10.1007/978-1-0716-2819-5_8 36335496

[CR42] Mérot C, Oomen RA, Tigano A, Wellenreuther M (2020). A roadmap for understanding the evolutionary significance of structural genomic Variation. TrendsEcol Evol.

[CR43] Milesi P, Lenormand T, Lagneau C, Weill M, Labbé P (2016). Relating fitness to long-term environmental variations *in natura*. Mol Ecol.

[CR44] Milesi P, Weill M, Lenormand T, Labbé P (2017). Heterogeneous gene duplications can be adaptive because they permanently associate overdominant alleles. Evol Lett.

[CR45] Milesi P, Assogba BS, Atyame CM, Pocquet N, Berthomieu A, Unal S (2018). The evolutionary fate of heterogeneous gene duplications: A precarious overdominant equilibrium between environment, sublethality and complementation. Mol Ecol.

[CR46] Milesi P, Claret J-L, Unal S, Weill M, Labbé P (2022). Evolutionary trade-offs associated with copy number variations in resistance alleles in *Culex pipiens* mosquitoes. Parasites Vectors.

[CR47] Namias A, Sahlin K, Makoundou P, Bonnici I, Sicard M, Belkhir K, Weill M (2023). Nanopore sequencing enables multigenic family reconstruction. Comput Struct Biotechnol J.

[CR48] N’Guessan R, Darriet F, Guillet P, Carnevale P, Traore-Lamizana M, Corbel V (2003). Resistance to carbosulfan in *Anopheles gambiae* from Ivory Coast, based on reduced sensitivity of acetylcholinesterase. Med Vet Entomol.

[CR49] Otto SP (2018). Adaptation, speciation and extinction in the Anthropocene. Proc R Soc B.

[CR50] Patterson EL, Pettinga DJ, Ravet K, Neve P, Gaines TA (2018). Glyphosate resistance and EPSPS gene duplication: convergent evolution in multiple plant species. J Hered.

[CR51] Reams AB, Kofoid E, Savageau M, Roth JR (2010). Duplication frequency in a population of *Salmonella enterica* rapidly approaches steady state with or without recombination. Genetics.

[CR52] Remnant EJ, Good RT, Schmidt JM, Lumb C, Robin C, Daborn PJ, Batterham P (2013). Gene duplication in the major insecticide target site, *Rdl*, in *Drosophila melanogaster*. Proc Natl Acad Sci USA.

[CR53] Schrider DR, Hahn MW (2010). Gene copy-number polymorphism in nature. Proc R Soc B Biol Sci.

[CR54] Schrider DR, Houle D, Lynch M, Hahn MW (2013). Rates and genomic consequences of spontaneous mutational events in *Drosophila melanogaster*. Genetics.

[CR55] Schumacher M, Camp S, Maulet Y, Newton M, MacPhee-Quigley K, Taylor SS (1986). Primary structure of *Torpedo californica* acetylcholinesterase deduced from its cDNA sequence. Nature.

[CR56] Scott JA, Brogdon WG, Collins FH (1993). Identification of single specimens of the *Anopheles gambiae* complex by the polymerase chain reaction. Am J Trop Med Hyg.

[CR57] Tamura K, Stecher G, Sudhir Kumar S (2021). MEGA11: molecular evolutionary genetics analysis version 11. Mol Biol Evol.

[CR58] Weetman D, Mitchell SN, Wilding CS, Birks DP, Yawson AE, Essandoh J (2015). Contemporary evolution of resistance at the major insecticide target site gene *Ace‐1* by mutation and copy number variation in the malaria mosquito *Anopheles gambiae*. Mol Ecol.

[CR59] Weill M, Lutfalla G, Mogensen K, Chandre F, Berthomieu A, Berticat C (2003). Insecticide resistance in mosquito vectors. Nature.

[CR60] Weill M, Malcolm C, Chandre F, Mogensen K, Berthomieu A, Marquine M, Raymond M (2004). The unique mutation in *ace-1* giving high insecticide resistance is easily detectable in mosquito vectors. Insect Mol Biol.

[CR61] Wellenreuther M, Mérot C, Berdan E, Bernatchez L (2019). Going beyond SNPs: the role of structural genomic variants in adaptive evolution and species diversification. Mol Ecol.

[CR62] Wickham H, Chang W, Wickham MH (2016). Package ‘ggplot2’. Create elegant data visualisations using the grammar of graphics. Version.

[CR63] Xu S, Li L, Luo X, Chen M, Tang W, Zhan L (2022). Ggtree: a serialized data object for visualization of a phylogenetic tree and annotation data. iMeta.

