# Supplementary material for: Despite structural identity, ace-1 heterogenous duplication resistance alleles are quite diverse in Anopheles mosquitoes
Source: Heredity (Edinb). 2024 Jan 27;132(4):179–91. doi: 10.1038/s41437-024-00670-9 (PMC10997782; doi:10.1038/s41437-024-00670-9)

**Supporting information Figure 1. A) Expected cluster sizes in a random draw, and B) observed occurrence distribution of the S and D haplotypes.**

A) To test our assumption that D(S) sequences could be recognized because they would be part of larger cluster than single-copy S alleles, we computed the distribution of the expected number of identical sequences in a random draw of 56 sequences (2 per 28 diploid individuals) out of the 26 different S sequences that composed our dataset (9 D(S) and 17 single-copy S), over 100,000 iterations. The probability of observing a given number of sequences in a cluster (*n_obs_*) is given by 1-(the corresponding quantile in the simulated distribution).

Solid lines represent the expected number of identical sequences for the first, second and third largest clusters (black, dark grey, light grey, resp.) over 100,000 iterations (see text). The dashed-dotted lines are the observed cluster sizes for D_1_, D_2_ and D_3_ (blue, brown and green respectively). Both D_1_ (*n*_obs_ = 8, *p* < 0.001) and D_2_ (*n*_obs_ = 11, *p* < 0.001) clusters were significantly larger than expected, whereas three-sequence clusters are expected in random draws (*p* = 0.89). Thus, apart from the independently-confirmed D_3_ allele (see text), D_4_(S), D_5_(S) and D_6_(S) identification remains tentative.

B) Distribution of number of occurrences per haplotype in S and D alleles recovered from the 28 individuals.


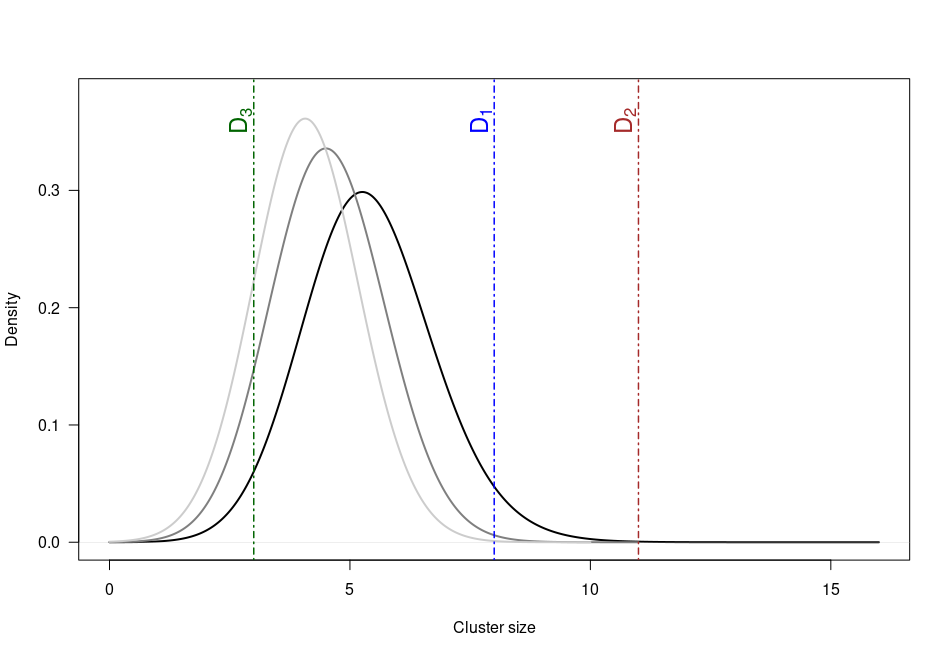
A) B)


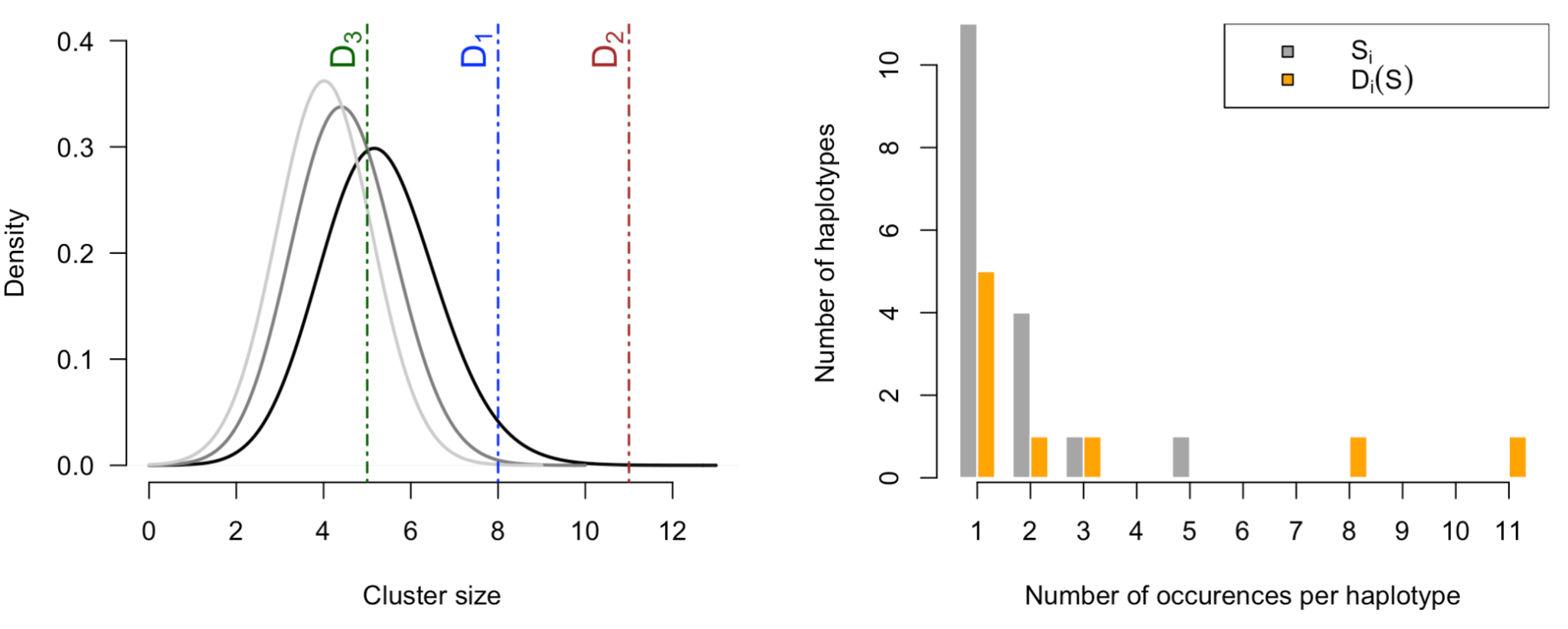


**Supporting information Figure 2.**  **Heterogeneous allele structure.**

In each graph, we present the variation of the standardized per-base depth of coverage (*pb*DoC*_std_,* with 1 being the mean *pb*DoC calculated over the whole chromosome) along the chromosomal region of interest (absiss, from 3.4 to 3.7 MB along the chromosome 2R). Each dot is the mean *pb*DoC*_std_* calculated every 100 bases (bin size) over 500-base sliding windows. The purple dashed lines represent the amplicon limits of the D_1_ and R^x^ alleles (Assogba et al. 2018); the cyan lines represent the *ace-1* gene location.


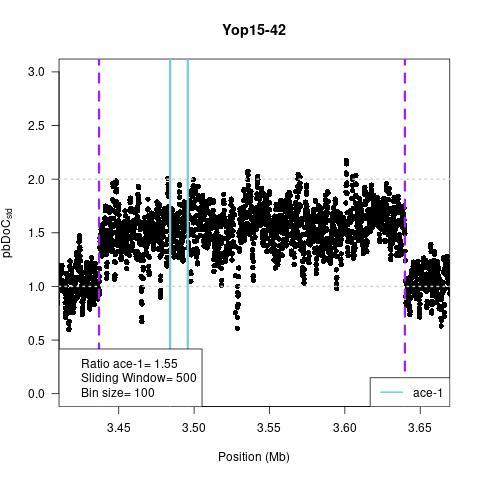

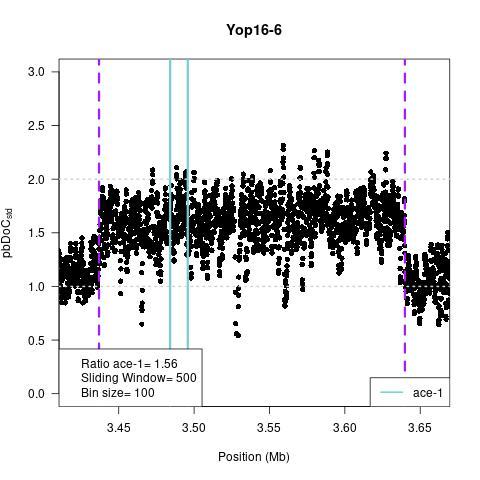

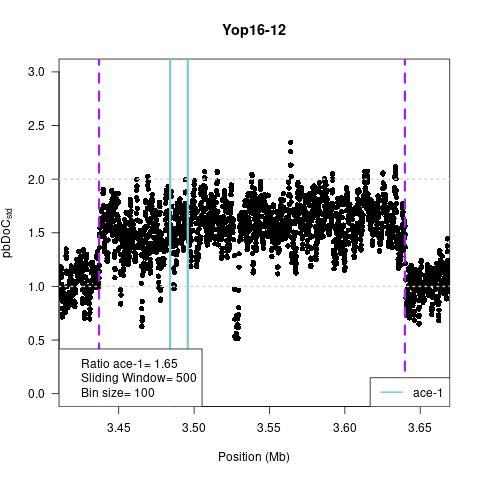

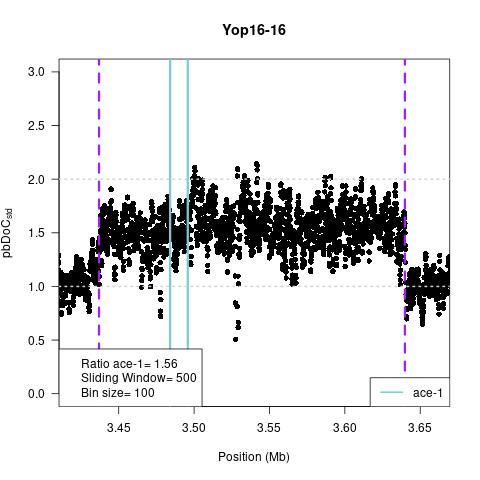

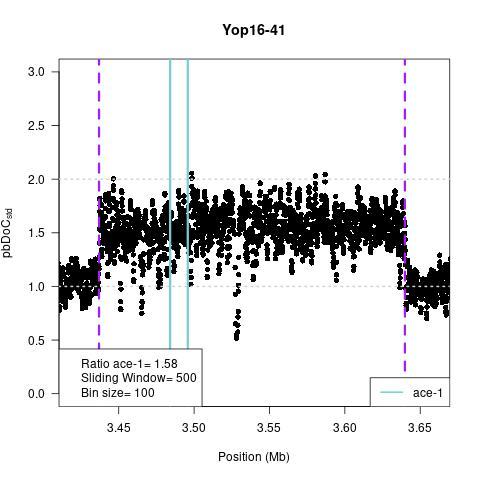

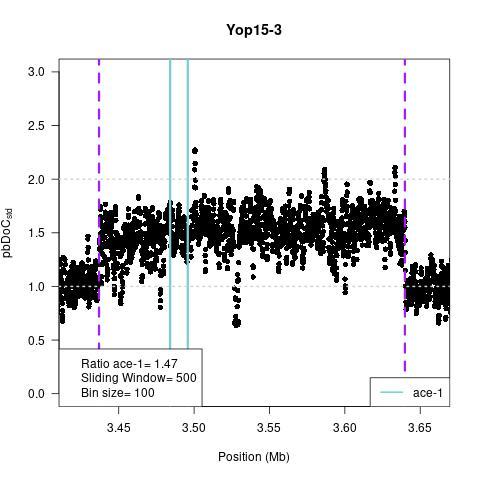


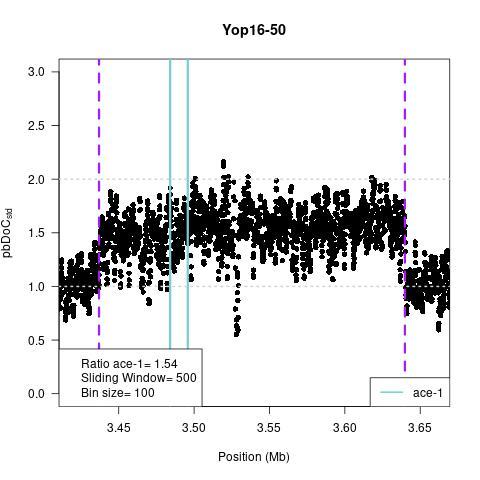

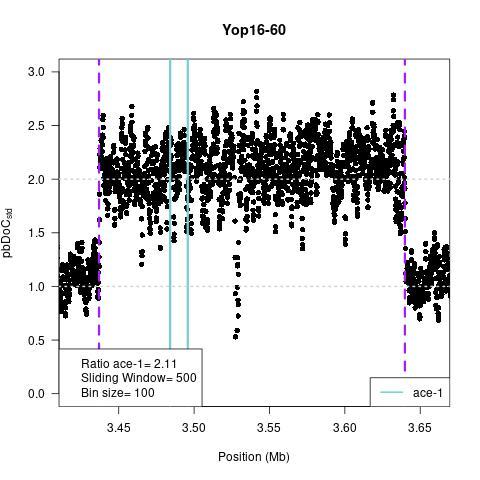


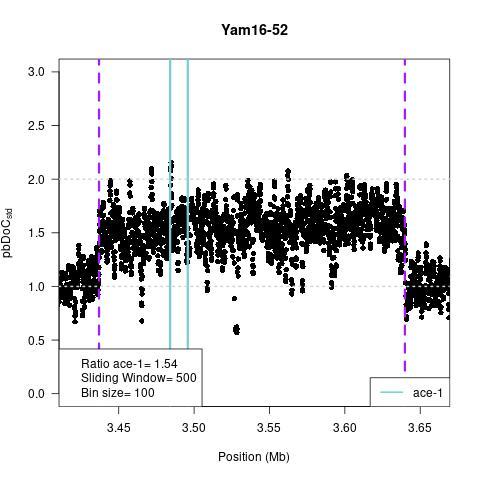

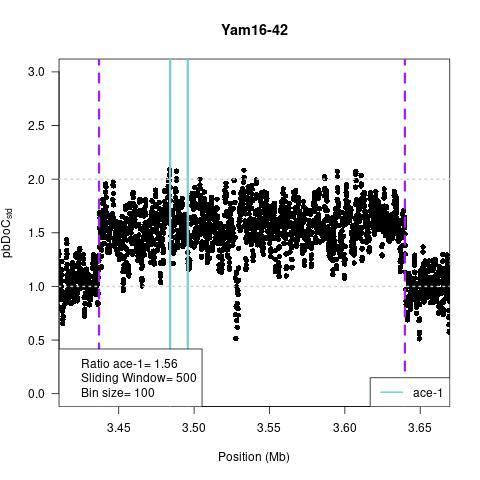

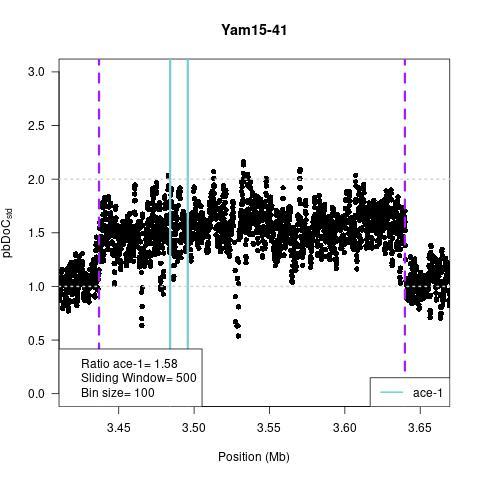

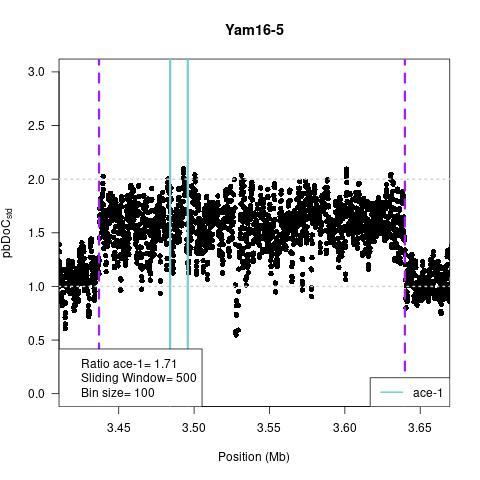


**Supporting information Figure 3: R, S and D allelic frequencies estimated in Yamoussoukro and Yopougon.**

Data from Assogba et al. 2018 (years 2012 to 2016), and from 2019 (this study) were used. We estimated the allelic frequencies considering a 3-allele model (all D alleles are considered together, Tab. 1 Model A) through a maximum likelihood approach (see also *Estimation of duplicated genotype frequencies* in Materials); they are shown in stacked plots with bars corresponding to upper and lower support limits (≈95% confidence intervals).


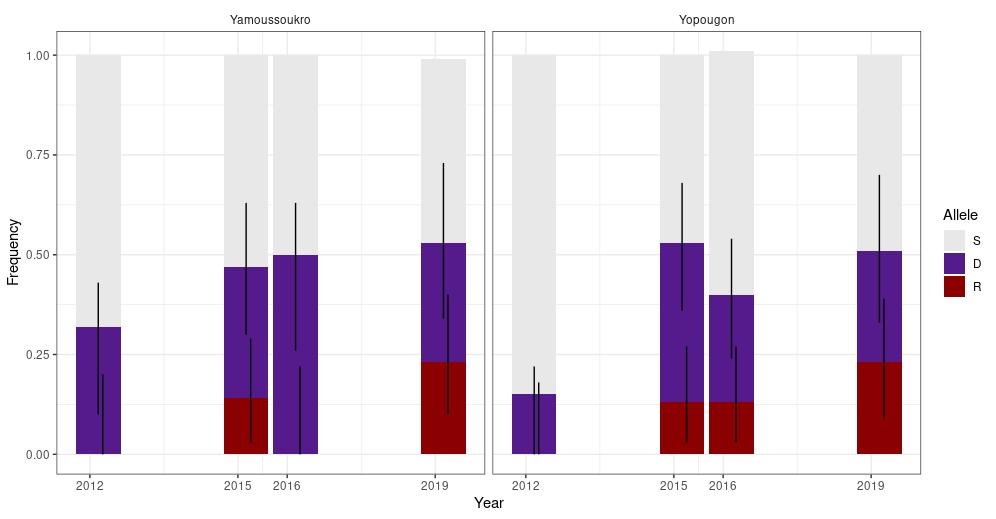

Supplement: Supplementary file 1 — Supplementary figures [file 41437_2024_670_MOESM1_ESM.docx]
