# Supplementary material for: Despite structural identity, ace-1 heterogenous duplication resistance alleles are quite diverse in Anopheles mosquitoes
Source: Heredity (Edinb). 2024 Jan 27;132(4):179–91. doi: 10.1038/s41437-024-00670-9 (PMC10997782; doi:10.1038/s41437-024-00670-9)
Supplement: Supplementary file 2 — Supplementary tables [file 41437_2024_670_MOESM2_ESM.docx]

**Supporting information Table 1. Molecular tests.**

Various PCR were used for identifying the species (Species) and the molecular form (Form) of the analysed individuals, as well as their *ace-1* phenotype, whether they carry the D_1_ allele (D_1_), and whether they carry a duplicated allele sharing the same junction between amplicons as D_1_ and R^x^ alleles (Junction). The primers used and the reaction characteristics are indicated.

| **PCR** | **Primers** | **Annealing temperature** |
| --- | --- | --- |
| **Species** | **- *Anopheles gambiae* AG** 5’ - CTG GTT TGG TCG GCA CGT TT - 3’  **- *Anopheles arabiensis* AA** - 5’ - AAG TGT CCT TCT CCA TCC TA - 3’  **- *Anopheles melas* AM** - 5’ - AAG TGT CCT TCT CCA TCC TA - 3’  **- Universal UN** - 5’ - CTG TGC CCC TTC CTC GAT GT - 3’ | 56°C |
| **Form** | **- 6.1a** 5’ - TCGCCTTAGACCTTGCGTTA - 3’.  **- 6.1b** 5 - CGCTTCAAGAATTCGAGATAC - 3’ | 52°C |
| ***ace-1***  **phenotype** | **- AgEx3univdir** 5’ - GAT CGT GGA CAC CGT GTT CG - 3’  **- AgEx3univrev** 5’ - AGG ATG GCC CGC TGG AAC AG - 3’ | 57°C |
| **D_1_** | **- AgEx3univdir** 5’ - GAT CGT GGA CAC CGT GTT CG - 3’  **- AgEx4rev** 5’ - TCG CTG CAT CTG CTG TCC GCC CT -3’ | 57°C |
| **Junction** | **- Agduplispedir2** 5’ - CTC TTA AGG TGG CGT TGT TCC - 3’  **- Agduplisperev1** 5’ - TTC CGC ACA AAA GGT TGG GCA - 3’ | 60°C |

**Supporting information Table 2. Summary of the different protocols applied to each sample of our study.**

For each individual, we summarised whether its *ace-1* haplotypes were determined through TA-cloning/Sanger sequencing and/or long-read sequencing (Nanopore). We also indicated those whose whole genome was sequenced (Illumina) (see Materials).

| **Sample** | **TA-cloning/**  **Sanger** | **Nanopore** | **Whole genome Illumina** |
| --- | --- | --- | --- |
| Yam12_12 | Yes | No | No |
| Yam12_25 | Yes | No | No |
| Yam12_39 | Yes | No | No |
| Yam12_55 | Yes | No | No |
| Yam12_56 | Yes | No | No |
| Yam15_41 | No | Yes | Yes |
| Yam16_42 | Yes | Yes | Yes |
| Yam16_52 | No | Yes | Yes |
| Yam16_5 | Yes | Yes | Yes |
| Yam19_11 | Yes | No | No |
| Yam19_14 | Yes | No | No |
| Yam19_39 | Yes | No | No |
| Yam19_6 | Yes | No | No |
| Yop12_43 | Yes | No | No |
| Yop12_45 | Yes | No | No |
| Yop12_54 | Yes | No | No |
| Yop15_3 | No | Yes | Yes |
| Yop15_42 | Yes | Yes | Yes |
| Yop15_49 | No | Yes | No |
| Yop16_12 | Yes | Yes | Yes |
| Yop16_16 | Yes | No | Yes |
| Yop16_1 | Yes | Yes | No |
| Yop16_41 | No | Yes | Yes |
| Yop16_50 | No | Yes | Yes |
| Yop16_60 | No | Yes | Yes |
| Yop16_6 | Yes | No | Yes |
| Yop19_24 | Yes | No | No |
| Yop19_5 | Yes | No | No |

**Supporting Information Table 3. Sequences dataset from previously published studies.**

The different sequences used in this study are indicated with their SRA accession number when available.

| **name** | **SRA_accession** | **name** | **SRA_accession** |
| --- | --- | --- | --- |
| Yam12_12 | PRJNA971118 | GhaaF4b_wt | KP165384.1 |
| Yam12_25 | PRJNA971118 | GhaaF4a_wt | KP165383.1 |
| Yam12_39 | PRJNA971118 | GhaaF1b_wt | KP165382.1 |
| Yam12_55 | PRJNA971118 | GhaaF1a_wt | KP165381.1 |
| Yam12_56 | PRJNA971118 | GhaaE1b_wt | KP165380.1 |
| Yam15_41 | PRJNA971118 | GhaaE1a_wt | KP165379.1 |
| Yam16_42 | PRJNA971118 | GhaaD1b_wt | KP165378.1 |
| Yam16_52 | PRJNA971118 | GhaaD1a_wt | KP165377.1 |
| Yam16_5 | PRJNA971118 | GhaaC1b_wt | KP165376.1 |
| Yam19_11 | PRJNA971118 | GhaaC1a_wt | KP165375.1 |
| Yam19_14 | PRJNA971118 | GhaaB2b_wt | KP165374.1 |
| Yam19_39 | PRJNA971118 | GhaaB2a_wt | KP165373.1 |
| Yam19_6 | PRJNA971118 | GhaaM_F2b | KP165362.1 |
| Yop12_43 | PRJNA971118 | GhaaM_F2a | KP165361.1 |
| Yop12_45 | PRJNA971118 | GhanaD3b_wt | KP165342.1 |
| Yop12_54 | PRJNA971118 | GhanaD3a_wt | KP165341.1 |
| Yop15_3 | PRJNA971118 | GhanaC3b_wt | KP165340.1 |
| Yop15_42 | PRJNA971118 | GhanaC3a_wt | KP165339.1 |
| Yop15_49 | PRJNA971118 | GhanaB3b_wt | KP165338.1 |
| Yop16_12 | PRJNA971118 | GhanaB3a_wt | KP165337.1 |
| Yop16_16 | PRJNA971118 | GhanaA3b_wt | KP165336.1 |
| Yop16_1 | PRJNA971118 | GhanaA3c_wt | KP165335.1 |
| Yop16_41 | PRJNA971118 | GhanaA3a_wt | KP165334.1 |
| Yop16_50 | PRJNA971118 | GhanaA1b_wt | KP165333.1 |
| Yop16_60 | PRJNA971118 | GhanaA1a_wt | KP165332.1 |
| Yop16_6 | PRJNA971118 |  |  |
| Yop19_24 | PRJNA971118 |  |  |
| Yop19_5 | PRJNA971118 |  |  |

**Supporting information Table 4. Probable genotypes of D-carrying individuals.**

The probable genotype of the 28 individuals identified as carrying at least one D allele, *i.e.* the triple-peak individuals, has been inferred from the phylogram (Fig. 2) and the genomic analyses, as described in Results. Each individual carried two S copies (S1 and S2, randomly assigned), one being D_i_(S) and the other one being either a single-copy S allele, or another D_j_(S) copy (“?” is used when undetermined). Each D allele is coloured as in Fig. 2. The different single-copy S alleles are identified as S_i_, where *i* (*i*= A to Q) indicates the haplotype. Whole-genome-Illumina-sequenced individuals are indicated with a “*”.

| **Individual** | **Copy S1** | **Copy S2** |
| --- | --- | --- |
| Yam12-12 | D_3_(S) | S_A_ |
| Yam12-25 | D_2_(S) | S_B_ |
| Yam12-39 | S_C_ | D_2_(S) |
| Yam12-55 | D_3_(S) | S_D_ |
| Yam12-56 | S_E_ | D_2_(S) |
| Yam15-41* | D_7_(S)? (S_F_) | D_7_(S)? (S_F_) |
| Yam16-42* | D_8_(S)? (S_G_) | D_8_(S)? (S_G_) |
| Yam16-5* | D_4_(S) | S_H_ |
| Yam16-52* | S_A_ | D_4_(S) |
| Yam19-11 | D_1_(S) | S_I_ |
| Yam19-14 | D_1_(S) | S_J_ |
| Yam19-39 | D_1_(S) | S_I_ |
| Yam19-6 | D_1_(S) | S_K_ |
| Yop12-43 | S_H_ | D_1_(S) |
| Yop12-45 | S_L_ | D_5_(S) |
| Yop12-54 | S_L_ | D_1_(S) |
| Yop15-3* | D_9_(S)? (S_M_) | D_9_(S)? (S_M_) |
| Yop15-42* | D_2_(S) | S_H_ |
| Yop15-49 | S_N_ | D_6_(S) |
| Yop16-1 | D_1_(S) | S_D_ |
| Yop16-12* | S_O_ | D_2_(S) |
| Yop16-16* | D_2_(S) | S_I_ |
| Yop16-41* | D_2_(S) | S_I_ |
| Yop16-50* | D_2_(S) | S_N_ |
| Yop16-6* | S_I_ | D_2_(S) |
| Yop16-60* | D_2_(S) | D_3_(S) |
| Yop19-5 | S_P_ | D_1_(S) |
| Yop19-24 | D_2_(S) | S_Q_ |

**Supporting information Table 5. Inferring total, R and S copy numbers from genomic data for triple-pic individuals.**

For each individual, the depth of coverage (DOC) was analysed first for the ratio between *ace-1* mean DOC and the mean DOC over the whole chromosome 2R (mean *ace-1*/mean chrom), and the ratio between *ace-1* mean DOC and the mean DOC of the single-copy reference gene *ace-2* (mean *ace-1/*mean *ace-2*) were computed. The expected ratios depend on the total number of *ace-1* copies: 1, 1.5, 2 and 2.5 respectively for 2, 3, 4 and 5 copies; the deduced number of *ace-1* copies is thus indicated.

We then analysed the number of reads *N* for R and S haplotypes at the position diagnostic (one base only) for resistance (S haplotypes carry a G, R haplotypes carry a A) found in the Illumina whole-genome sequencing, giving the frequency of the R haplotypes among all the reads covering this position (%R). This frequency is expected to be 0.5, 0.33 and 0.25 for frequencies corresponding respectively to 1R:1S (RS or DD), 1R:2S (DS) and 1R:3S (e.g. D alleles with multiple S copies).

The copy number for each haplotype (R or S) was deduced considering both the *ace-1* total copy number and the frequency of R haplotypes. When considering only the diagnostic base, some ratios are not compatible with the total copy number (bolded; NB: these individuals actually carry DS genotypes, *i.e*. 1R:2S; see text).

|  |  |  | ***ace-1*** |  |  | **diagnostic base** | | |  |
| --- | --- | --- | --- | --- | --- | --- | --- | --- | --- |
| **individual** |  | **mean *ace-1*/ mean chrom** | **mean *ace-1/* mean *ace-2*** | **deduced *ace-1* total copy number** |  | ***N***  **R reads** | ***N***  **S reads** | **%R** | **deduced number of haplotypes** |
| Yam15-41 |  | 1.58 | 1.49 | 3 |  | 15 | 29 | 0.34 | 1R:2S |
| Yam16-42 |  | 1.56 | 1.38 | 3 |  | 13 | 26 | 0.33 | 1R:2S |
| Yam16-5 |  | 1.71 | 1.47 | 3 |  | 14 | 25 | 0.36 | 1R:2S |
| Yam16-52 |  | 1.54 | 1.47 | 3 |  | 12 | 36 | **0.25** | not compatible |
| Yop15-3 |  | 1.47 | 1.46 | 3 |  | 14 | 26 | 0.35 | 1R:2S |
| Yop15-42 |  | 1.55 | 1.41 | 3 |  | 10 | 28 | **0.26** | not compatible |
| Yop16-12 |  | 1.65 | 1.81 | 3 |  | 18 | 31 | 0.37 | 1R:2S |
| Yop16-16 |  | 1.56 | 1.31 | 3 |  | 16 | 34 | 0.32 | 1R:2S |
| Yop16-41 |  | 1.58 | 1.54 | 3 |  | 16 | 31 | 0.34 | 1R:2S |
| Yop16-50 |  | 1.54 | 1.55 | 3 |  | 16 | 23 | **0.41** | not compatible |
| Yop16-6 |  | 1.61 | 1.55 | 3 |  | 12 | 25 | 0.32 | 1R:2S |
| Yop16-60 |  | 2.11 | 02.09 | 4 |  | 27 | 28 | 0.49 | 2R:2S |
